# *Salmonella*-based platform for efficient delivery of functional binding proteins to the cytosol

**DOI:** 10.1038/s42003-020-1072-4

**Published:** 2020-07-03

**Authors:** Antoine Chabloz, Jonas V. Schaefer, Ivona Kozieradzki, Shane J. F. Cronin, Daniel Strebinger, Francesca Macaluso, Jiri Wald, Terence H. Rabbitts, Andreas Plückthun, Thomas C. Marlovits, Josef M. Penninger

**Affiliations:** 1 IMBA, Institute of Molecular Biotechnology of the Austrian Academy of Sciences, Vienna, Austria; 2 Department of Medical Genetics, Life Science Institute, University of British Columbia, Vancouver, BC Canada; 3 Center for Structural Systems biology (CSSB), University Medical Center Hamburg-Eppendorf (UKE) and German Electron Synchrotron Centre (DESY), Hamburg, Germany; 4 Department of Biochemistry, University of Zürich, Zürich, Switzerland; 5 Weatherall Institute of Molecular Medicine, MRC Molecular Haematology Unit, University of Oxford, John Radcliffe Hospital, Oxford, United Kingdom; 6 Present Address: Novartis Institutes for Biomedical Research, Basel, Switzerland; 7 Present Address: Broad Institute of MIT and Harvard, Cambridge, MA United States

**Keywords:** Protein transport, Protein delivery, Cancer metabolism, Applied microbiology, Molecular engineering

## Abstract

Protein-based affinity reagents (like antibodies or alternative binding scaffolds) offer wide-ranging applications for basic research and therapeutic approaches. However, whereas small chemical molecules efficiently reach intracellular targets, the delivery of macromolecules into the cytosol of cells remains a major challenge; thus cytosolic applications of protein-based reagents are rather limited. Some pathogenic bacteria have evolved a conserved type III secretion system (T3SS) which allows the delivery of effector proteins into eukaryotic cells. Here, we enhance the T3SS of an avirulent strain of *Salmonella typhimurium* to reproducibly deliver multiple classes of recombinant proteins into eukaryotic cells. The efficacy of the system is probed with both DARPins and monobodies to functionally inhibit the paradigmatic and largely undruggable RAS signaling pathway. Thus, we develop a bacterial secretion system for potent cytosolic delivery of therapeutic macromolecules.

## Abstract (web summary)

To develop a bacterial secretion system for cytosolic delivery of therapeutic macromolecules, Chabloz et al. improve an “effectorless” *Salmonella* strain and combine it with a plasmid modified to boost the secretion of proteins of interest. With this system, they demonstrate efficient translocation of functional DARPins and monobodies into the cytosol of different eukaryotic cells lines and successfully block the paradigmatic RAS pathway.

## Introduction

Eukaryotic membranes are essential for cell homeostasis and therefore act as major barriers for macromolecules. Due to these fundamental functions, it is challenging to efficiently deliver proteins across the cell membranes to the cytosol of eukaryotic cells. The majority of the currently attempted cytosolic protein delivery systems rely on polycations (cell-penetrating peptides (CPPs)^1,2^ or supercharged proteins), which are believed to work through osmotic lysis of endosomes. However, this is an inefficient process which requires rather high protein concentrations^3^. Nanoparticles have also been used, employing a variety of mechanisms depending on their compositions, yet mainly relying on endosomal escape mechanisms^4,5^. In addition, bacterial toxins have the capability to translocate proteins through defined endosomal cell membrane pores^6–9^. Finally, certain pathogenic bacteria have developed molecular machines allowing the transport of specific proteins across plasma membranes in order to manipulate eukaryotic cells^10,11^.

In contrast to small molecules, which usually require extensive medicinal chemistry to achieve and demonstrate specificity, recombinant binding proteins such as Designed Ankyrin Repeat Proteins (DARPins)^12^, nanobodies^13^ or monobodies^14^ can readily be selected for high affinities and specificities against almost any target. However, these macromolecules cannot readily access the cytosolic space which limits their utility for fundamental mechanistic studies and their analytical and therapeutic potential. Recombinant binders have previously been delivered, with various degrees of success, to intracellular compartments, using CPPs^15,16^, nanoparticles^17^ and bacterial toxins^8,18,19^, with marked differences in cytosolic delivery efficiencies between cell types^7^. In contrast, the T3SS has been used successfully for the delivery of many different proteins, including nanobodies, in multiple cell types and organisms^20–31^. However, to our knowledge, no study has been published to rigorously verify the functionality of delivered DARPins, once they have reached the cytosol. For other recombinant binding proteins with inhibitory function against essential pathways, only two reports have described delivery of binders via a toxin system^18,19^, while another study reported some activity of a binder coupled to a polyarginine tail^16^. Due to the limitations in cytosolic deliveries, the functionality of such synthetic proteins is mainly studied via transfection, stable intracellular expression or microinjection. For example, these methods were utilized to study the RAS pathway with multiple DARPins and a monobody targeting the RAS protein as well as DARPins targeting upstream (ErbB) or downstream (ERK) effector proteins^32–37^.

In the present study, we enhance the T3SS of an avirulent strain of *Salmonella enterica serovar typhimurium* (*S. typhimurium*) for effective cytosolic delivery of large amounts of synthetic binding proteins into multiple cell types. We also show that such delivery allows functional inhibition of RAS signaling.

## Results

### Enhancement of a bacterial system for protein delivery

**Fig. 1:**
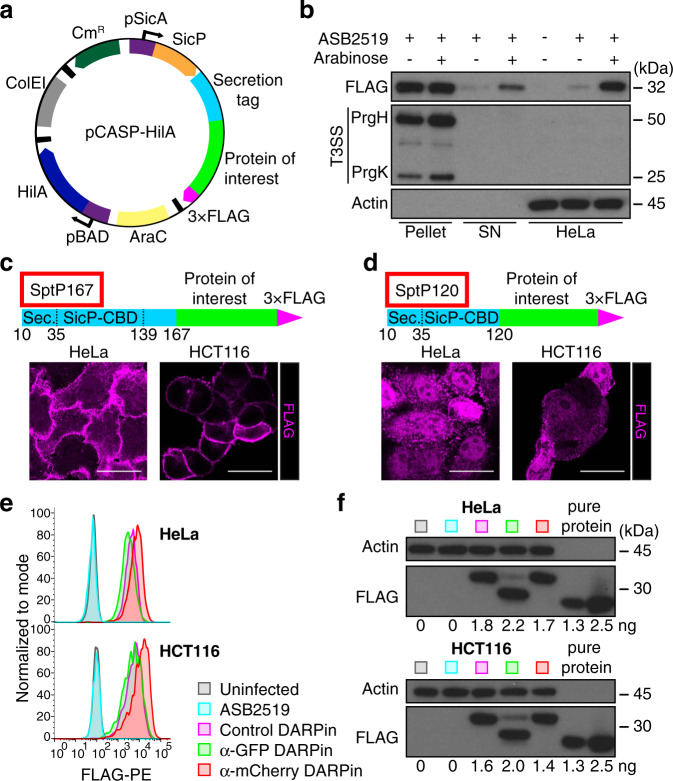
Improvement of the T3SS into an efficient cytosolic delivery system. **a** Schematic representation of the pCASP-HilA secretion system plasmid. The translation of the SptP specific chaperone SicP (in orange) and the fusion protein from **c** or **d** are coupled on a single mRNA^65^ and regulated by the *Salmonella* pSicA promoter. The main regulator of SPI-1 genes HilA is under the regulation of an arabinose-inducible promoter (pBAD). Black boxes represent transcriptional termination and purple boxes with arrows represent promoters. Cm^R^: chloramphenicol resistance, ColEI: origin of replication, AraC: Arabinose operon regulatory protein. **b** Anti-FLAG tag western blot of bacterial pellets and supernatants (SN) after 4 h induction of *Salmonella* ASB2519 with or without arabinose in the last 2 h, and HeLa cells infected with those *Salmonella* for 1 h at a MOI of 100 in the presence or absence of arabinose. Anti-needle complex (T3SS) and anti-Actin blots serve as control for the presence of *Salmonella* and eukaryotic cells, respectively. **c, d** (Top) Schematic of the transferred SptP167 and SptP120 3 × FLAG-tagged fusion recombinant proteins. Secretion signal (Sec.), SicP-chaperone binding domain (SicP-CBD) and amino acid numbers that mark the beginning and end of each SptP domain are indicated^39^. Representative anti-FLAG-immunostaining images of anti-GFP (α-GFP) FLAG-tagged DARPins transferred into HeLa and HCT116 cells using the SptP167 (**c**) or SptP120 (**d**) secretion tags (1 h infection at a MOI of 100). Scale bars represent 25 µm. **e** Flow cytometry analysis of 3 different FLAG-tagged SptP120-DARPins (E3_5 control, α-GFP 3G124 and α-mCherry 3m160 DARPins) transferred into HeLa and HCT116 cells following 1 h infection at a MOI of 100. Uninfected cells and *Salmonella* ASB2519 with an empty pCASP-HilA vector (ASB2519) served as negative controls. Experiments were repeated in three biological replicates showing comparable results. **f** Quantitative anti-FLAG tag western blot of HeLa and HCT116 cells from the same experiment as in **e**. Numbers below the blots show quantification of the respective transferred SptP120-DARPin fusion proteins, based on 1.3 and 2.5 ng of a purified 3×FLAG protein (pure protein - RuvC) loaded on the same blot. Uncropped blots can be found in Supplementary Fig. 6.

To induce secretion of a synthetic protein from *Salmonella*, we took advantage of the previously developed pCASP plasmid system^38^. In this system, the expression of recombinant proteins is under the control of the pSicA promoter and thus co-regulated with *Salmonella* pathogenicity island-1 (SPI-1) gene expression. Recombinant proteins were tagged with a 3 × FLAG tag at their C-termini and at their N-termini with the first 167 amino acids of the natural *Salmonella* effector protein SptP (SptP167) for secretion (Fig. 1a). SptP167 contains both a secretion signal and a chaperone binding domain (CBD)^39^ — two domains that have previously been shown to be sufficient and necessary for secretion by the T3SS^40^. Moreover, we mutated another known pathogenic T3SS-1 effector (SopA) and the T3SS-2 indispensable protein SsaK in the “effectorless” *S. typhimurium* strain SB2519 (generously provided by the Galán laboratory^41^) to bioengineer a novel avirulent strain called ASB2519 (Supplementary Table 1), carrying a disrupted T3SS-2 to block its capacity to replicate intracellularly in case of phagocytosis^42^.

To boost T3SS-1 expression in *Salmonella* and thereby permitting increased transfer of proteins, we inserted the known key regulator of SPI-1 genes, HilA^43–45^, into the pCASP vector under an arabinose-inducible promoter (pBAD). Using this novel pCASP-HilA vector (Fig. 1a) and following the induction with arabinose, a marked increase in both proteins secretion into the supernatant (SN) from ASB2519 *Salmonella* cultures as well as cytoplasmic transfer of proteins into HeLa cells (human cervix adenocarcinoma cells) was achieved (Fig. 1b).

Next HeLa cells and HCT116 cells (human colorectal carcinoma cells) were infected with this avirulent ASB2519 strain of *S. typhimurium*, carrying the pCASP-SptP167-3G124-HilA plasmid to express and deliver the anti-GFP DARPin 3G124^46^ fused to the SptP167 secretion tag (the infection strategy is outlined in Supplementary Fig. 1). We observed effective transfer of the anti-GFP DARPin; however, the transferred binder predominantly localized at the plasma membrane of both tested eukaryotic cell types (Fig. 1c). Such membrane localization was also seen using a different DARPin (called E3_5, which has no defined binding specificity^47^, Supplementary Fig. 2), suggesting that the addition of SptP167 results in mis-localization of the delivered affinity reagents. For this reason, and based on the crystal structure of SptP-CBD bound to a dimer of its specific chaperone SicP^39^, we designed a shortened SptP secretion tag termed SptP120 (terminated at threonine-120 encompassing the first three, but excluding the forth, interaction domains of SptP-CBD with the SicP dimer^39^). Importantly, in contrast to the membrane mis-localization found for full-length SptP167-fused DARPins, this SptP120 secretion tag resulted in intracellular delivery of SptP120-fused DARPins at the predicted cytoplasmic and nuclear localizations (Fig. 1d).

Moreover, using flow cytometry, we analyzed transfer efficiency of the anti-GFP DARPin into both HeLa as well as HCT116 cells. Importantly, the modified system reproducibly delivered DARPins into >95% of the cells (Fig. 1e). These data were confirmed using two additional DARPin binders (Fig. 1e), the control DARPin E3_5 as well as an anti-mCherry binder (3m160)^46^. Of note, we have also tested two shorter secretion tags either composed of the 35 or 92 first amino acids of SptP (encompassing none or two interaction domains of SptP-CBD with the SicP dimer^39^, respectively) which resulted in a complete block of secretion for a protein fused to SptP35 and a significantly reduced efficiency of cytosolic transfer for the SptP92-fused protein (Supplementary Fig. 3). Intracellular delivery of DARPins fused to the SptP120 secretion tag was further demonstrated by quantitative western blotting, indicating that ~1.9 ng and 1.7 ng of each DARPin could be delivered into 75,000 HeLa and 150,000 HCT116 cells, respectively, after a 1-h infection at a multiplicity of infection (MOI) of 100 (Fig. 1f). This equals a concentration of 188 nM in HeLa cells and 126 nM in HCT116 cells, with measured single cell volumes of 4.2 pL and 2.8 pL, respectively. Of note, intracellular delivery of the SptP120 DARPins did not show any apparent cytotoxicity in HeLa and HCT116 cells (Supplementary Fig. 4). Thus, to our knowledge, this novel *S. typhimurium* T3SS delivery system enables highly efficient cytosolic transfer of macromolecular DARPins. To confirm T3SS-1 specific translocation of DARPins, we performed the same secretion and transfer experiments as mentioned above either with the ASB2519 strain carrying a disrupted T3SS-1 (by knocking-out the essential gene, *PrgH*^48^) or using a pCASP-HilA vector lacking the SptP120 secretion tag fused to the DARPin (Supplementary Fig. 5). As expected, we see no secretion, nor transfer of proteins to target cells when the T3SS-1 is compromised and removal of SptP120 disrupted expression of the synthetic protein.

### Cytoplasmic delivery of functional DARPins

**Fig. 2:**
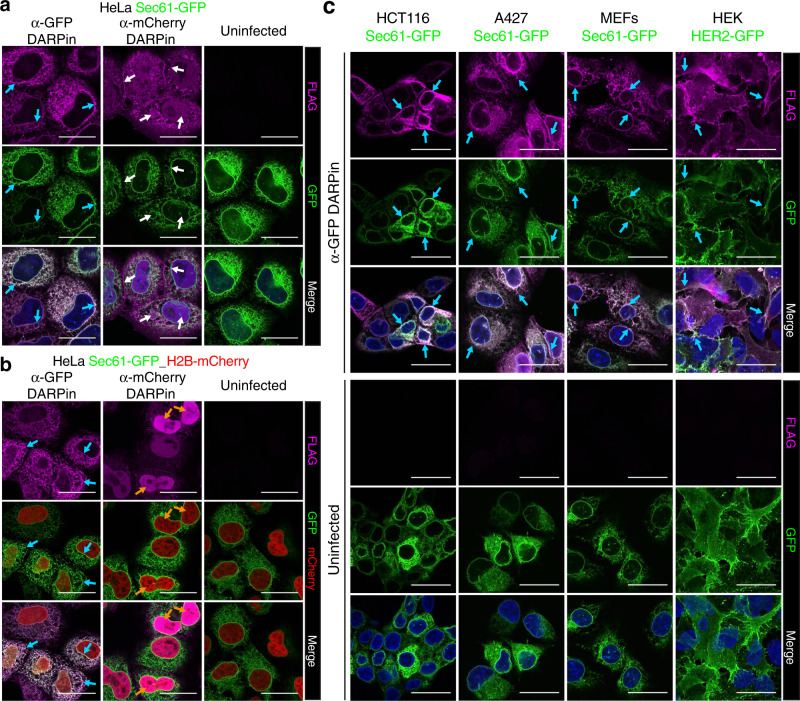
Delivery of functional DARPins into multiple cell types by *Salmonella* ASB2519. **a, b** Representative anti-FLAG-immunostaining images (magenta, upper panels) of anti-GFP (α-GFP) and α-mCherry FLAG-tagged SptP120-DARPins transferred into HeLa cells (1 h infection at a MOI of 100). Experiments were performed in HeLa cells expressing (**a**) Sec61-GFP or (**b**) both Sec61-GFP and H2B-mCherry. GFP (green) and mCherry (red) expression and merged images are also shown. **c** Representative anti-FLAG-immunostainings of α-GFP FLAG-tagged SptP120-DARPin transferred into the indicated human and mouse cell types expressing Sec61-GFP and HER2-GFP. Experiments were repeated in three biological replicates showing comparable results. Uninfected samples served as negative controls. Scale bars represent 25 µm. Blue and orange arrows indicate colocalization in the cytoplasm and in nuclei, respectively. White arrows show that the anti-FLAG signal does not co-localize with GFP using the α-mCherry DARPin.

Effector proteins need to unfold to pass through the narrow needle complex channel of the T3SS and, once transferred, the proteins must refold in the cytoplasm of the eukaryotic cell to be functional^49^. Our data so far have demonstrated efficient intracellular delivery of DARPins, indicating that they are properly unfolded for secretion, despite the known high stability of these proteins^47,50^. Once inside the eukaryotic cells, DARPins need to refold to be functional and bind their targets. To validate regain of function after transfer, we delivered the previously used anti-GFP DARPin into HeLa cells constitutively overexpressing either the endoplasmic reticulum (ER) transport protein Sec61 subunit beta fused with GFP on its cytoplasmic N-terminus (Sec61-GFP) or the nuclear histone protein H2B fused to GFP (H2B-GFP). When we probed for the transferred anti-GFP DARPin, we indeed observed its expected co-localization (detected by its FLAG-tag) with GFP at the cytoplasmic membrane of the ER for Sec61-GFP or in the nucleus for H2B-GFP, respectively (Fig. 2a and Supplementary Fig. 7a). As a control, the anti-mCherry DARPin 3m160^46^ did not co-localize with GFP (Fig. 2a). To ensure that the system generally can transfer functional DARPins, we next delivered them into HeLa cells overexpressing the cytoskeleton protein alpha-Tubulin-mCherry. As can be seen in Supplementary Fig. 7b, the anti-mCherry DARPin, but not the anti-GFP binder co-localized with the mCherry-labeled cytoskeleton protein alpha-Tubulin. Finally, we delivered both DARPins into HeLa cells co-expressing both Sec61-GFP and H2B-mCherry. The anti-GFP DARPin co-localized with GFP on the ER membranes while the anti-mCherry DARPin targeted the mCherry-fusion protein in the nucleus (Fig. 2b), confirming specificity of the transferred DARPins.

As the efficiency of most other intracellular delivery system greatly varies and largely depends on the cell type used^7^, we next tested whether our new system would enable the delivery of functional DAPRins into multiple cell types. For this, we generated HCT116, A427 (human lung carcinoma cells), and immortalized mouse embryonic fibroblasts (MEFs) that constitutively express Sec61-GFP. Moreover, we tested HER2-GFP-expressing human HEK293 embryonic kidney cells, where GFP is fused to the C-terminus and is thus located on the cytosolic side of the plasma membrane. In all five different cell lines from two different species (mouse and human), we observed delivery of the anti-GFP DARPin and its correct co-localization with the overexpressed GFP-fusion target (Fig. 2c). Furthermore, in HEK293 cells co-localization of the anti-GFP DARPin with the membrane protein HER2 could be detected (Fig. 2c). Of note, although DARPins generally are biophysically stable proteins, we detected reduced levels of the bacteria-delivered proteins within all the tested cells at ~3 h post-infection. However, addition of the proteasome inhibitor bortezomib (BZB)^51^ rescued the levels of the intracellular delivered fusion-proteins (Supplementary Figs. 7c, d and 8). Importantly, BZB treatment had no apparent effect on the specific co-localization of DARPins to their respective targets (Supplementary Figs. 7d and 8). These data show DARPins can be delivered into multiple cell types and that the transferred DARPins are functional and co-localize with their specific target proteins.

### Delivery of recombinant binders to inhibit RAS signaling

**Fig. 3:**
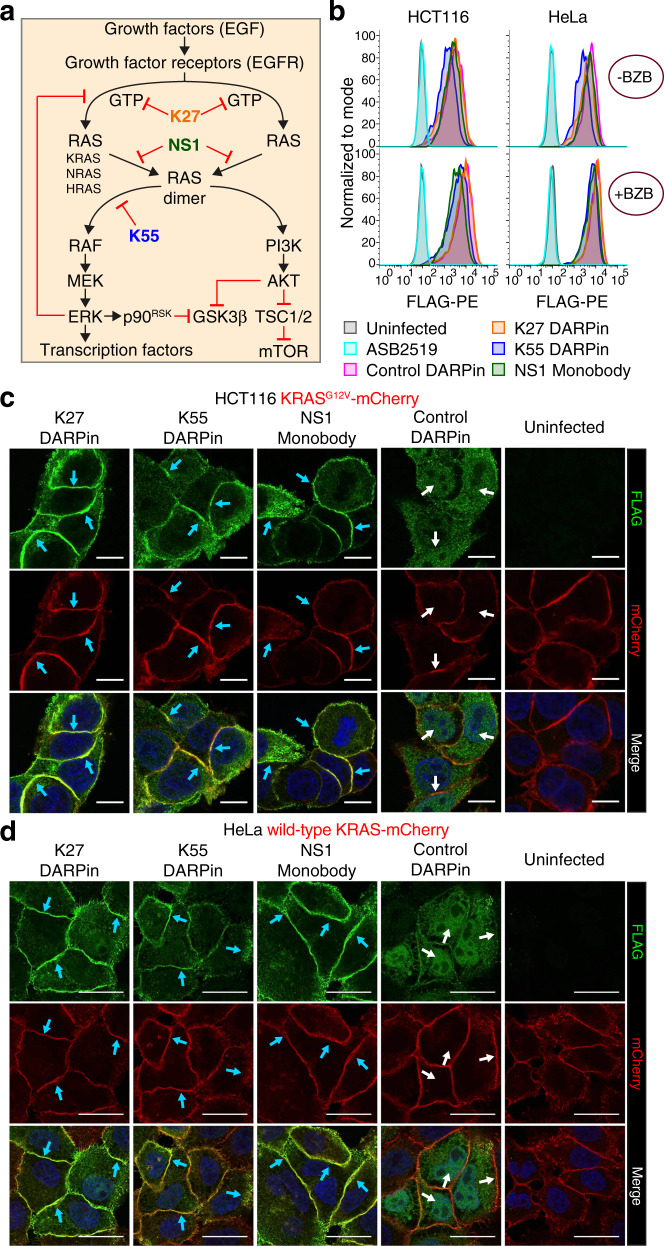
Synthetic RAS inhibitory proteins are efficiently delivered into cells and co-localize with overexpressed mutated or wild-type KRAS proteins. **a** Simplified RAS pathway overview with the DARPins (K27 and K55) and monobody (NS1) used to block RAS activation (modified from www.cancer.gov). Black arrows indicate activation and red lines show inhibition. **b** Flow cytometry analysis of the FLAG-tagged SptP120-control DARPin and SptP120-anti-RAS inhibitors transferred into HCT116 and HeLa cells (1 h infection at a MOI of 100), in the absence (upper histograms) or presence (lower histograms) of the proteasome inhibitor bortezomib (BZB; 50 nM). Uninfected cells and cells infected with *Salmonella* ASB2519 with an empty pCASP-HilA vector (ASB2519) served as negative controls. Representative anti-FLAG-immunostainings (green) of FLAG-tagged SptP120-anti-RAS inhibitors transferred into HCT116 (**c**) and HeLa (**d**) cells expressing KRAS^G12V^ or wild-type KRAS-mCherry. All samples were treated with BZB followed by 10 min incubation in fresh medium without (**c**) or with (**d**) EGF (20 ng per mL) stimulation. Experiments were repeated in two biological replicates showing comparable results. Uninfected cells and SptP120-control DARPin served as negative controls. Scale bars are (**c**) 10 µm or (**d**) 25 µm. Blue arrows indicate colocalization. White arrows show that the anti-FLAG signal does not co-localize with mCherry using the control DARPin.

Having performed proof-of-concept experiments with the anti-GFP and anti-mCherry DARPins, we next aimed to use our system to deliver synthetic proteins to inhibit RAS activity. RAS is a key pathway of multiple growth factor signaling cascades and one of the most frequently mutated genes in human cancer^52,53^. With the exception of the *KRAS*^*G12C*^ mutation, for which a covalent inhibitor has been described^54^, all other oncogenic KRAS mutants still remain undruggable^55^. Two DARPins (K27 and K55)^32^ and a monobody (NS1)^33^ have been recently developed to block RAS and overexpression of these inhibitory proteins (using >24 h transfections and stable integrations) has been reported to downregulate activation of the RAS pathway in cell lines harboring *RAS* mutations, yet by different modes of action: K27 inhibits RAS-GTP nucleotide exchange and K55 out-competes the downstream effector RAF while NS1 prevents RAS dimerization (Fig. 3a).

To test whether our bacterially-delivered affinity reagents can inhibit RAS signaling, HCT116 and HeLa cells were infected for 1 h with the avirulent *Salmonella* strain ASB2519 delivering either K27, K55, NS1 or the control DARPin E3_5. We observed efficient delivery of all DARPins and also the monobody into HCT116 as well as HeLa cells (Fig. 3b). Proteasome inhibition again resulted in higher levels of the delivered proteins (Fig. 3b). To confirm that the bacterially-delivered DARPins and the NS1 monobody were binding to their targets, we delivered them in HCT116 cells that were engineered to stably express an oncogenic KRAS^G12V^ fused to mCherry at its N-terminus as well as in HeLa cells expressing mCherry-tagged wild-type KRAS. Both DARPins and the NS1 monobody co-localized with overexpressed mCherry-tagged oncogenic KRAS (Fig. 3c) as well as with EGF-activated wild-type KRAS (Fig. 3d). Thus, all synthetic RAS inhibitory proteins tested can be efficiently transferred and faithfully co-localize to their specific target.

**Fig. 4:**
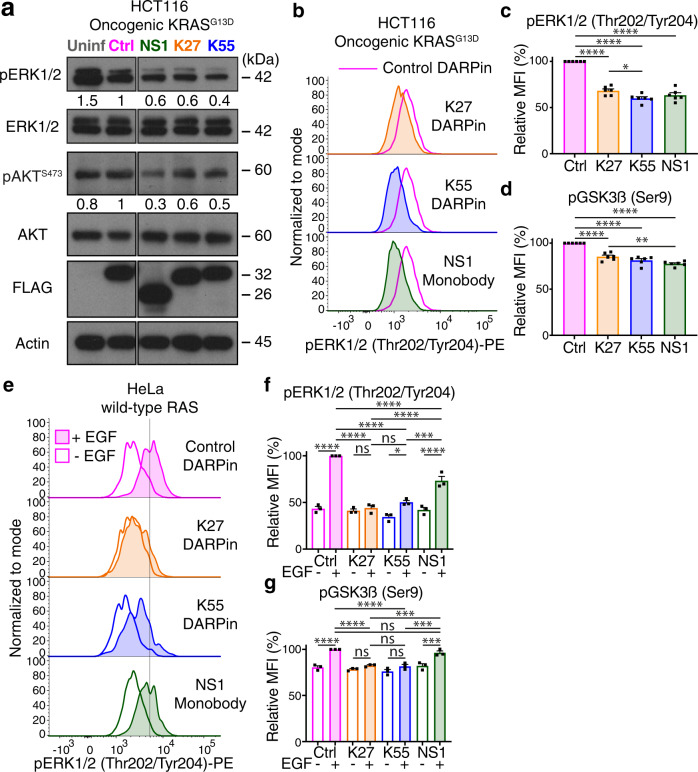
T3SS-delivered RAS inhibitors downregulate RAS signaling. **a** Western blot analysis of uninfected HCT116 cells (Uninf) and HCT116 cells infected with *Salmonella* ASB2519 (1 h, MOI of 100) delivering a FLAG-tagged SptP120-control (Ctrl) DARPin and the SptP120-anti-RAS inhibitory DARPins K27 and K55 or the RAS blocking monobody NS1. Anti-FLAG detection served as the transfer control and total anti-ERK1/2, AKT and Actin as loading controls. Numbers below the pERK1/2 and pAKT blots detected with anti-pERK1/2 (Thr202/Tyr204) and anti-pAKT (Ser473) antibodies represent the quantified fraction relative to the SptP120-control DARPin (Ctrl), normalized to total ERK1/2 and AKT, respectively. Uncropped blots can be found in Supplementary Fig. 10. **b** Flow cytometric measurements of ERK1/2 phosphorylation in HCT116 cells upon FLAG-positive delivery of the indicated SptP120-anti-RAS binders. Data were analyzed 10 min post-infection in the presence of bortezomib (BZB; 50 nM). Pink lines indicate baseline ERK1/2 phosphorylation levels upon delivery of the SptP120-control DARPin. Relative median fluorescence intensities (MFI) of ERK1/2 (**c**) and GSK3β (**d**) phosphorylation compared to the SptP120-control DARPin (Ctrl) treated cells and using the same experimental setup as in **b**. **e** Flow cytometric analysis of ERK1/2 phosphorylation in HeLa cells upon FLAG-positive delivery of the indicated SptP120-anti-RAS binders and a control DARPin. Data were analyzed 10 min post-infection in the presence of bortezomib (BZB) without (empty) or with EGF (20 ng per mL) stimulation (filled). Relative median fluorescence intensities (MFI) of ERK1/2 (**f**) and GSK3β (**g**) phosphorylation compared to the SptP120-control DARPin (Ctrl) treated cells with EGF and using the same experimental setup as in **e**. Data represent the mean ± SEM of six (**c**, **d**) or three (**f**, **g**) biological replicates. Individual data points are shown. Statistical analysis was performed using a one-way ANOVA with Tukey’s multiple comparisons test (****P **<** 0.0001; ***P **<** 0.001; **P **<** 0.01; *P **<** 0.05; ns not significant; only relevant comparisons are indicated for (**f**) and (**g**)).

HCT116 cells harbor a G13D activating mutation of *KRAS* (KRAS^G13D^) resulting in a constitutively activated KRAS pathway^56^. Delivery of K27 and K55 DARPins as well as the NS1 monobody into HCT116 cells caused a markedly reduced phosphorylation of both ERK1/2 (pERK1/2 Thr202/Tyr204) and AKT (pAKT Ser473) (Fig. 4a), two major signaling events downstream of active RAS (Fig. 3a). Inhibition of the constitutively active RAS pathway in HCT116 cells was confirmed in multiple independent experiments by intracellular flow cytometry to detect active phosphorylated ERK1/2 at 10 min (Fig. 4b, c) or 3 h (Supplementary Fig. 9a) post-infection. Moreover, we noted reduced phosphorylation of the ERK1/2 and AKT downstream mediator GSK3β (pGSK3β Ser9) (see Fig. 3a) by flow cytometry following intracellular delivery of both DARPins and the monobody (Fig. 4d and Supplementary Fig. 9b). Of note, since the delivery of the control DARPin also resulted in a minor downregulation of ERK1/2 activation, we always used this as a control for the flow cytometry analyses.

To examine the inhibitory effects of these proteins on EGF-mediated induction of wild-type RAS, we used HeLa cells which carry wild-type *RAS* alleles. Bacterially-delivered anti-RAS DARPins again markedly abrogated EGFR-induced ERK1/2 and GSK3β phosphorylation, as determined by intracellular immunostaining (Fig. 4e–g). Importantly, whereas the NS1 monobody inhibited RAS activation to a similar level as both DARPins in HCT116 cells carrying an activating *KRAS*^*G13D*^ mutation, the NS1 monobody showed a significantly reduced capacity to block EGFR-induced activation of wild-type RAS (Fig. 4). These data show that our system allows for the efficient transfer of DARPins as well as monobodies into human cancer cells. Importantly, such bacterially-delivered synthetic binding proteins can rapidly (~1 h) and significantly block the activation of oncogenic KRAS as well as growth factor-induced wild-type RAS.

## Discussion

Reliable methods exist to rapidly develop highly selective recombinant affinity reagents that can theoretically target any protein, including key players of intracellular disease pathways^12,14,57^. Simultaneously, major efforts have been made to establish systems for efficient intracellular delivery of macromolecular proteins^3,6,8,15–21,24–26^, as reliable delivery systems would open yet unexplored opportunities for basic research as well as drug discovery. However, most of the available systems are still based on endosomal escape and thus rather inefficient and highly variable between various cell types^7^, compounded by sometimes inadequate measurements of cytoplasmic localization^3^.

Here we report an avirulent *S. typhimurium*-based delivery system for recombinant proteins, specifically for DARPins and monobodies, which exhibits several advantages. First, the genetically manipulated *S. typhimurium* ASB2519 strain lacks all the known natural pathogenic effectors and is thereby unable to enter into eukaryotic cells^41^. Moreover, if phagocytosed, ASB2519 shouldn’t be able to replicate within eukaryotic cells as we disrupted the indispensable T3SS-2 protein SsaK^58^. Of note, infections with our newly developed *S. typhimurium* ASB2519 strain are highly reproducible. Second, the proteins can be stably and highly expressed in *S. typhimurium* and hence do not require any purification or formulation steps. Third, only a single cloning step is required to introduce a new protein for delivery and we discovered a secretion tag that allows for accurate localization of the synthetic proteins within the cells (Figs. 1, 2). Forth, since >95% of cells receive the transferred protein, kinetic studies of e.g., inhibition of defined signaling pathways can thus be performed. Finally, our system reliably and efficiently delivers functional proteins into multiple different cell types from different species, where they can reach their respective targets in the cytosol and nucleus.

Importantly, we were able to deliver DARPins and monobodies, two structurally diverse proteins, indicating that our system can be utilized for the intracellular delivery of different proteins families. Whether larger proteins can also be efficiently transferred needs to be determined in future experiments. One limitation that needs to be addressed in additional experiments is the detected degradation of transferred proteins, which can be avoided using a proteasome inhibitor. Whether ubiquitination of the transferred proteins targets them for proteasomal degradation, maybe even as a function of the fusion to SptP120^59^, or whether degradation is mediated through another pathway needs to be examined. Our system might well be suited to unravel such a question and could possibly become a useful tool to study the fundamental protein degradation biology and design non-degradable synthetic proteins.

To demonstrate the power of our system, we used DARPins and monobodies that specifically inhibit RAS, one of the paradigmatic signaling pathways in normal physiology and cancer. RAS is mutated in ~25% of human cancers^52^ and is thus a key oncogene for malignant transformation. Inhibition of different constitutively active RAS family molecules is therefore a prime focus of interest to develop effective anti-cancer drugs; unfortunately, major challenges persist that need to be overcome in order to accomplish such inhibition, one of them being efficient delivery. Using our system, we were indeed able to deliver three different RAS-inhibitory synthetic proteins that, most importantly, could effectively block oncogenic as well as growth factor (EGF)-induced RAS signaling. These three RAS-blockers target different molecular interactions: the DARPin K27 inhibits RAS-GTP nucleotide exchange, the DARPin K55 out-competes the downstream effector RAF, and the monobody NS1 prevents RAS dimerization (see Fig. 3a for a scheme). All of these synthetic proteins abrogated ERK1/2 and GSK3β signaling downstream of oncogenic KRAS in HCT116 cells. By contrast, in HeLa cells possessing wild-type RAS, K27 and K55 nearly completely blocked EGF-induced RAS signaling, whereas such inhibition was markedly less detectable following transfer of NS1. Although such studies need to be extended to additional protein inhibitors and other cell types, these data suggest that our system could be used to probe the precise molecular mechanisms of RAS activation and possibly to develop synthetic protein-based drugs that selectively inhibit oncogenic, but not wild-type RAS. Of note, our finding parallels a recent study showing that DARPins targeting the allosteric lobe of KRAS - the same as our NS1 monobody - block mutant KRAS, but not wild-type RAS when transfected^35^.

In addition to the T3SS of *S. typhimurium*, other bacteria are being explored for intracellular protein delivery^20–31^. For instance, *Pseudomonas aeruginosa* has been used to deliver transcription factors and enzymes, promoting cell differentiation or gene editing^25,26,28,31^ while a *Yersinia enterocolitica*-based system could transfer various proteins, including two different pro-apoptotic proteins inducing cell death in vitro and in vivo^20^. Moreover, *S. typhimurium* and its T3SS have been used as vaccination vectors for the delivery of multiple different antigens in mice^22,23,27,29^. Building on previous exquisite 3D cryo-EM structures and functional information on the T3SS of *S. typhimurium*^48^, we were able to enhance the system’s potential as an efficient and reliable cytosolic delivery platform for macromolecules. Moreover, we utilized the HilA transcriptional regulator in *S. typhimurium*^43–45^ that allowed us to not only increase the expression of the synthetic proteins, but also to drive enhanced T3SS expression, thereby significantly boosting the transfer of the synthetic proteins. Thus, our system enables delivery of large quantities of proteins directly into the cytoplasm. In our T3SS delivery system, recombinant proteins of the DARPin and monobody type unfold to move through the needle complex and subsequently refold within the target cells to faithfully bind to their respective targets. Importantly, as shown for the RAS inhibitors, they can functionally block signaling.

Overall, our bacterial delivery system offers a screening platform to validate and discover intracellular synthetic protein inhibitors that nowadays can be readily and specifically engineered to target protein-protein interactions or block enzymatic activities. Our cytosolic delivery system also allows direct and rapid functional mapping of synthetic proteins to develop potent macromolecular inhibitors of yet undruggable targets to explore novel therapeutic avenues.

## Methods

### Bacterial strains

The *Salmonella* strain SB2519^41^ was transformed with the lambda red plasmid^60^ and subsequently used to generate mutants using transformation with polymerase chain reaction (PCR) fragments amplified on the pKD3 plasmid^61^ with primers ACp255 + ACp256 for SopA (another known pathogenic T3SS-1 effector still present in SB2519) and primers ACp258 + ACp259 for the T3SS-2 indispensable protein SsaK. To generate the *Salmonella* strain ASB2519, mutations were subsequently transferred in a recipient *Salmonella* SB2519 strain by P22 phage transduction as described^62^ and the resistance cassette flanked by FRT sites was flipped via transformation with the heat-sensitive pCP20 plasmid^63^. Similarly, PCR fragment for PrgH knock-out was generated with primers ACp367 + ACp368 on pKD3 and transformed in the *Salmonella* strain ASB2519 carrying the lambda red plasmid. PrgH mutations were then transferred via P22 phage transduction in a recipient *Salmonella* ASB2519 strain and resistance cassette flipped with pCP20. *Salmonella* strains and primers used in this study are listed in Supplementary Tables 1 and 2, respectively.

### Gibson assembly

Primers for Gibson assembly were designed to have a length of 44 nucleotides, with 22 nucleotides for amplifying the sequence of interest and 22 nucleotides as annealing overhangs. Gradient-temperature PCR was performed with in-house Phusion polymerase at annealing temperatures calculated for the respective primers. PCR products were run on a 1% agarose gel in TAE buffer and purified with QIAquick Gel Extraction Kit (#28704, Qiagen). Equimolar amounts of vector and insert(s) were added to 10 µL of in-house Gibson assembly master mix in a total volume of 20 µL. The reaction was carried out for 1 h at 50 °C and incubated for 5 min on ice prior to transformation of chemically competent *E. coli* DH5α produced in house. Plasmid DNA was prepared from positive clones and all constructs were confirmed by Sanger sequencing.

### Generation of pCASP-HilA plasmids and growth conditions

Secretion pCASP-HilA plasmids were obtained by Gibson assembly. The vector pCASP^38^ was linearized by restriction digest of its single AvrII site, and a fragment coding for an arabinose-inducible HilA construct was amplified by PCR with primers ACp100 + ACp101 from the pSB667 plasmid^45^. Subsequently, the pCASP-HilA vector was amplified with forward primer ACp133 and reverse primer ACp134 for SptP167, ACp147 for SptP120, ACp146 for SptP92 or ACp145 for SptP35 secretion tags. The inserts with the corresponding 22-nucleotide overhangs were ordered as gBlock fragments (Integrated DNA Technologies) and cloned with Gibson assembly. Plasmid pCASP-3G124-HilA was built via Gibson assembly of two PCR fragments amplified with primer pairs (ACp133 + ACp292) and (ACp248 + AC p370) on pCASP-SptP120-3G124-HilA. All plasmids are listed in Supplementary Table 3 and maps and sequences are deposited at addgene. Strains carrying the pCASP-HilA plasmid were grown in LB medium (Sigma) containing 25 µg per mL chloramphenicol (Sigma) at 37 °C with aeration.

### Preparation of electrocompetent *Salmonella*

*S. typhimurium* were grown until reaching an optical density of 0.5 measured at a wavelength of 600 nm (OD600). After incubation on ice for 30 min, two washes with ultra-high pure water and two washes with 1x PBS (Gibco) containing 10% glycerol (AppliChem) were performed at 4 °C. The electrocompetent *S. typhimurium* were resuspended in 1x PBS supplemented with 10% glycerol, aliquoted and immediately snap-frozen in liquid nitrogen and stored at −80 °C. Electrocompetent *S. typhimurium* were electroporated with 50 ng of plasmid DNA in a pre-chilled electroporation cuvette (#1652086, BioRad) at 1800 V with an electroporator (220 V 940000017, Eppendorf). Super optimal broth with catabolite repression (SOC) medium (ThermoFisher) was added just after electroporation and *S. typhimurium* were grown for 1 h at 37 °C before plating on agar plates containing the respective antibiotic.

### *Salmonella* secretion and infection assays

*Salmonella* were grown (directly from stable glycerol stocks) overnight at 37 °C in LB with chloramphenicol at 220 rpm in a shaking incubator. The next morning, cultures were diluted 1 in 10 (1/10) in 5 mL of LB containing chloramphenicol and supplemented with 0.3 M NaCl to induce *Salmonella* pathogenicity island-1 (SPI-1) gene expression. After a 2 h growth period, arabinose was added to a final concentration of 0.012% (weight/volume) to induce the expression of HilA and subsequent secretion of recombinant proteins fused to SptP120 or SptP167 in the following 2 h (Supplementary Fig. 1). For secretion assays, cultures of *Salmonella* ASB2519 were grown for a total of 4 h and then directly added to eukaryotic cells at a multiplicity of infection (MOI) of 100 for infections assays. Infections were carried out for 1 h in the respective eukaryotic cell culture medium supplemented with 25 µg per mL chloramphenicol and 0.012% (w/v) arabinose. After 1 h of infection, *Salmonella* ASB2519 were removed and eukaryotic cells washed once with 1x PBS and further incubated without *Salmonella* ASB2519 for 10 min in cell culture medium either without or with 20 ng per mL EGF or for 3 h with 200 µg per mL gentamycin and during the last 10 min without or with 20 ng per mL EGF. When indicated, the proteasome inhibitor bortezomib (BZB) (Abcam) was applied at 50 nM for 30 min prior to infection and sustained throughout the entire experiment (Supplementary Fig. 1).

The cytotoxicity of our *Salmonella* strain ASB2519 delivering SptP120 fused to DARPins was analyzed via lactate dehydrogenase cytotoxicity assay LDH (Supplementary Fig. 4). LDH release from infected and uninfected eukaryotic cells was measured with the CytoTox 96 Non-Radioactive Cytotoxicity Assay (G1780, Promega) following the manufacturer’s instructions. The relative amount of LDH release was calculated as follows: percentage released LDH (sample) = (sample − medium background)/(maximum LDH − medium background) ×100.

### Protein purification

As quantification control for the western blot analysis, 3×FLAG-RuvC protein was expressed in the *E. coli* strain BL21(DE3) harboring the plasmid pET-52b(+) carrying the coding sequence corresponding to RuvC derived from *S. typhimurium*. Cells were grown in LB broth supplemented with 100 µg per mL ampicillin at 37 °C to an OD600 of 0.6. Expression of RuvC was induced by the addition of 1 mM IPTG and cultures were further incubated at 37 °C for 3 h. Subsequently, cells were collected by centrifugation, washed in wash buffer [20 mM Tris-HCl (pH 8), 20 mM NaCl and 1 mM EDTA], resuspended in lysis buffer [100 mM Tris-HCl, pH 8, 500 mM NaCl and 10% glycerol] and stored at −80 °C. Prior to purification cells were lysed by sonication and resulting suspension was centrifuged at 37,000 rpm for 1 h at 4 °C. Cell-free lysate was applied onto a HisTrap HP column equilibrated with buffer 1 [100 mM Tris-HCl (pH 8), 10% glycerol and 500 mM NaCl] and bound proteins were eluted with a linear gradient of 10 mM → 500 mM imidazole. Peak fractions were pooled and dialyzed against buffer 1. The protein pool was loaded onto a Superdex 200 column equilibrated with buffer 2 (100 mM Tris pH 8, 500 mM NaCl, 1 mM EDTA, 0.5 mM DTT, 15% glycerol). The peak containing RuvC protein eluted at 15 mL, was collected and stored at −80 °C. The purity of RuvC was >90%, as judged by SDS-PAGE followed by staining with Coomassie blue (Supplementary Fig. 11).

### Western blot analysis

*Salmonella* from a 5 mL culture (also used in the secretion assays) were pelleted at 4 °C and 6000 × *g* for 15 min. The bacterial culture supernatants were harvested and 0.2 µm filtered (“supernatant secretion samples”) while the *Salmonella* pellets were resuspended in 5 mL of 1x PBS and mixed in a 1/1 ratio with 87% (volume/volume) glycerol (“expression samples”). Secretion and expression samples were analyzed without a concentration step. For western blot analysis, eukaryotic cells were infected as described in Supplementary Fig. 1 in 6-well plates. At the end of the experiment, cells were washed once with 1x PBS; to harvest cells, Accutase (A1110501, ThermoFisher) was added for 10 min at 37 °C. After centrifugation at 4 °C and 2000 × *g* for 2 min, cell pellets were immediately frozen in liquid nitrogen and stored at −80 °C. Cell pellets were thawed on ice and resuspended in 1x PBS with 0.002% digitonin (Sigma) and supplemented with protease inhibitor cocktail (cOmplete, Roche). Resuspended pellets were kept on ice for 5 min and centrifuged at 4 °C and 16,000 × *g* for 25 min. Since digitonin does not lyse bacterial membranes, supernatant containing only eukaryotic cells content was harvested and the total protein content determined by a Bradford protein assay (#5000001, BioRad). Samples were separated on a 4-12% gradient NuPAGE gel (NP0335BOX, ThermoFisher) and blotted on polyvinylidene difluoride (PVDF) membrane by wet transfer. PVDF membranes were probed with: mouse monoclonal M2 anti-FLAG (1/3000 dilution, F1804, Sigma), mouse anti-actin (1/1000 dilution, A5316, Sigma), rabbit anti-phospho-ERK1/2 (Thr202/Tyr204) (1/1000 dilution, #9101, Cell Signaling), rabbit anti-ERK1/2 (1/1000 dilution, #9102, Cell Signaling), rabbit anti-phospho-AKT (Ser473) (1/1000 dilution, #9271, Cell Signaling), rabbit anti-AKT (1/1000 dilution, #4685, Cell Signaling), or rabbit anti-T3SS (1/3000 dilution, in house generated) antibodies in milk or phosphoblocker (AKR-103-S, Cell Biolabs) diluted in washing buffer (1x TBS-0.1% Tween-20) overnight at 4 °C. Detection was performed with either secondary anti-mouse IgG (1/4000 dilution, W4021, Promega) or anti-rabbit IgG (1/4000 dilution, NA9340, Sigma) antibodies conjugated to horseradish peroxidase, and visualized using enhanced chemiluminescence (ECL Plus, #32132, ThermoFisher).

### Lentiviral vector production and infection

Lenti-mCherry-G12V-KRAS-IRES-Blast and Lenti-mCherry-WT-KRAS-IRES-Blast were obtained by Gibson assembly. The vectors were amplified by PCR with primers ACp326 + ACp327 from the Lenti-AcGFP-Sec61-IRES-Blast plasmid, the mCherry inserts with primers ACp328 + ACp329 from a plasmid containing mCherry, and the KRAS insert with primers ACp330 + ACp331 from wild-type or G12V-KRAS containing plasmids (gift from Boehringer Ingelheim). Lentiviral vectors were produced in 6-well plates as previously described^64^ by transfection of Lenti-X 293T cells with 4 µg of plasmid vector (Lenti-AcGFP-Sec61-IRES-Blast, Lenti-mCherry-G12V-KRAS-IRES-Blast or Lenti-mCherry-WT-KRAS-IRES-Blast), packaging plasmid pCMVR8.74 and vesicular stomatitis G producing plasmid at a ratio of 4:2:1. Culture medium was collected 24 h post-transfection, 0.2 µm filtered and directly used to infect recipient cells for 24 h. This procedure was repeated a second time 48 h post-transfection. Transduced cells were selected for blasticidin (ThermoFisher) resistance resulting in a mixed population of integration-positive cells expressing various level of either GFP-tagged Sec61 subunit beta or mCherry-tagged oncogenic (G12V) or wild-type KRAS.

### Cell culture

All cell lines used in this study are listed in Supplementary Table 4 and were grown in high glucose DMEM (Gibco) supplemented with 10% fetal bovine serum (FBS) (Gibco), non-essential amino acids, 2 mM L-glutamine (Sigma) and 25 mM HEPES (Gibco).

### Immunofluorescence

Cells were seeded on pre-treated (70% ethanol, 0.1% gelatin; 30 min) coverslips (#72196-12, EMS) in 24-well plates the day before the infection experiment. At the end of the infection experiment (Supplementary Fig. 1), cells were washed once with 1x PBS and subsequently fixed with 4% paraformaldehyde (PFA) (#28908, ThermoFisher) for 10 min at room temperature (RT) and permeabilized and blocked for 1 h at RT in blocking buffer (1x PBS supplemented with 5% FBS, 2% bovine serum albumin (BSA), 1% glycine and 0.2% Triton X-100). The specimens were stained with primary mouse monoclonal M2 anti-FLAG antibodies (1/1000 dilution, F1804, Sigma) overnight at 4 °C in a humid chamber, washed three times for 15 min at RT with washing buffer (1x PBS supplemented with 0.2% Triton X-100, 1% glycine) and stained with secondary Alexa 647-labeled anti-mouse IgG (1/500 dilution, ab150115, Abcam). Specimens were again washed three times for 15 min at RT and nuclei were counterstained with DAPI (1/2000 dilution, D3571, ThermoFisher). Finally, specimens were mounted onto cover glasses (SuperFrost Ultra Plus, ThermoFisher) with fluorescence mounting medium (S3023, Dako) and confocal images were taken using a Carl Zeiss LSM880 microscope with Airyscan.

### Flow cytometry

Cells were seeded in 96-well plates the day before the infection. Following the infection experiments (Supplementary Fig. 1), cells were washed once with 1x PBS and Accutase was added for 10 min at 37 °C for harvesting. Cells were subsequently stained with the fixable viability dye eFluor^TM^ 780 (1/1000 dilution, 65-0865, ThermoFisher) for 15 min on ice and fixed with 4% PFA for 10 min at RT. Sample was permeabilized in blocking buffer for 30 min at RT and stained with primary mouse monoclonal M2 anti-FLAG antibody (1/1000 dilution, F1804, Sigma) followed by directly PE-labeled secondary anti-mouse IgG F(ab’)2 antibody (1/1000 dilution, 12-4010-87, ThermoFisher) in blocking buffer, each for 20 min at RT. For phosphorylation analyses, samples were fixed with BD Cytofix (#554655, BDbiosciences) for 15 min at 37 °C and permeabilized in Perm/Wash buffer (#557885, BDbiosciences) for 20 min at RT. Samples were stained with directly labeled FITC-anti-FLAG M2 (1/50 dilution, F4049, Sigma), PE-anti-phospho-ERK (Thr202/Tyr204) (1/100 dilution, #369506, biolegend) or Pacific Blue-anti-phospho-GSK3β (Ser9) (1/100 dilution, #14310, Cell signaling) for 1.5 h at RT or overnight at 4 °C in a humid chamber. Samples were analyzed with a FACS LSR Fortessa (BDbiosciences). To compare experiments performed on different days, we set the median fluorescence intensity (MFI) of cells receiving the control DARPin E3_5 (with EGF induction of the cells when needed) to 100% per condition and phosphorylation marker and subsequently calculated the relative percentage MFI under the different experimental conditions.

### Statistics and reproducibility

All values in this study are given as means ± SEM. Details of the statistical tests applied are stated in the figure legends. GraphPad Prism software was used to create bar graphs and perform statistical analyses. Data were analyzed by using a one-way ANOVA with Tukey’s multiple comparisons test, as indicated. *P* < 0.05 was considered as statistically significant.

### Reporting summary

Further information on research design is available in the Nature Research Reporting Summary linked to this article.

## Supplementary information

Supplementary Information

Supplementary Data 1

Supplementary Data 2

Supplementary Data 3

Supplementary Data 4

Description of Additional Supplementary Files

Reporting Summary

## Data Availability

The datasets generated and analyzed during the current study are available from the corresponding authors upon reasonable request. All plasmids have been deposited to addgene with unique identifiers (ID: 153322-153333, 153335, 153336 and 154009). Associated raw data for Fig. 4c, d, f, and g can be found as supplementary data.
