# Supplementary material for: Salmonella-based platform for efficient delivery of functional binding proteins to the cytosol
Source: Commun Biol. 2020 Jul 3;3:342. doi: 10.1038/s42003-020-1072-4 (PMC7335062; doi:10.1038/s42003-020-1072-4)
Supplement: Supplementary file 1 — Supplementary Information [file 42003_2020_1072_MOESM1_ESM.pdf]

Supplementary data files

Supplementary Figures

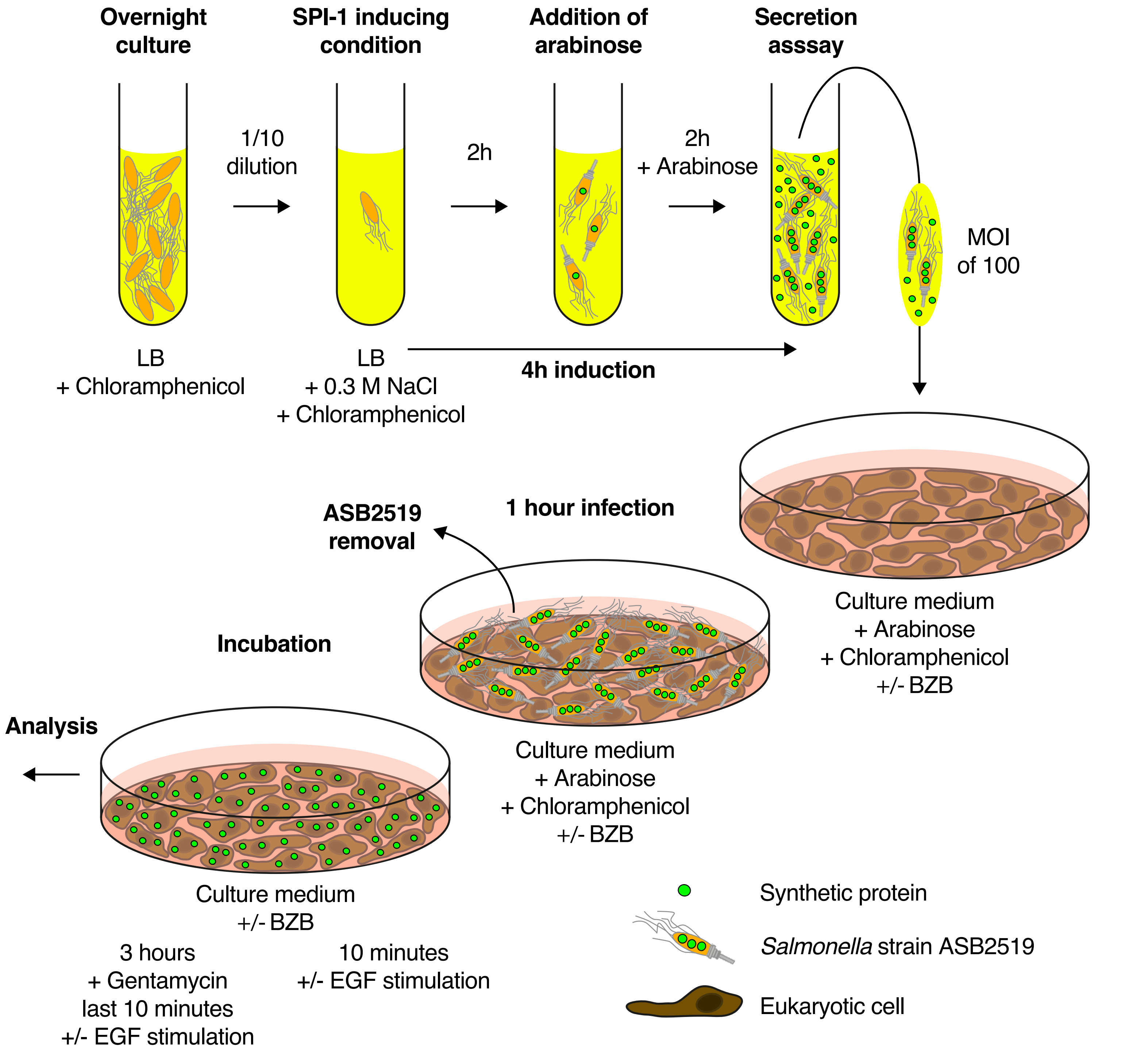

Supplementary Figure 1. Schematic overview of the Induction and infection procedures.
Salmonella ASB2519 were grown in lysogeny broth (LB) complemented with chloramphenicol overnight, followed by a 1 to 10 dilution in Salmonella pathogenicity island-1 (SPI-1)-inducing conditions (LB + 0.3 M NaCl). Expression of Salmonella ASB2519 SPI-1 T3SS genes and DARPin or monobody was first induced in high salt LB for 2 hours, followed by 2 additional hours of T3SS induction via the addition of arabinose (0.012%). After a total of 4 hours of induction, Salmonella ASB2519 were directly employed at a multiplicity of infection (MOI) of 100 for a 1-hour infection of eukaryotic cells in growth medium complemented with arabinose and chloramphenicol. After removal of Salmonella ASB2519, eukaryotic cells were either incubated for 10 minutes or 3 hours in growth medium with Gentamycin to avoid further proliferation and infection of remaining Salmonella ASB2519. In some experiments, bortezomib (BZB) and/or epidermal growth factor (EGF) were added (see Material and Methods).

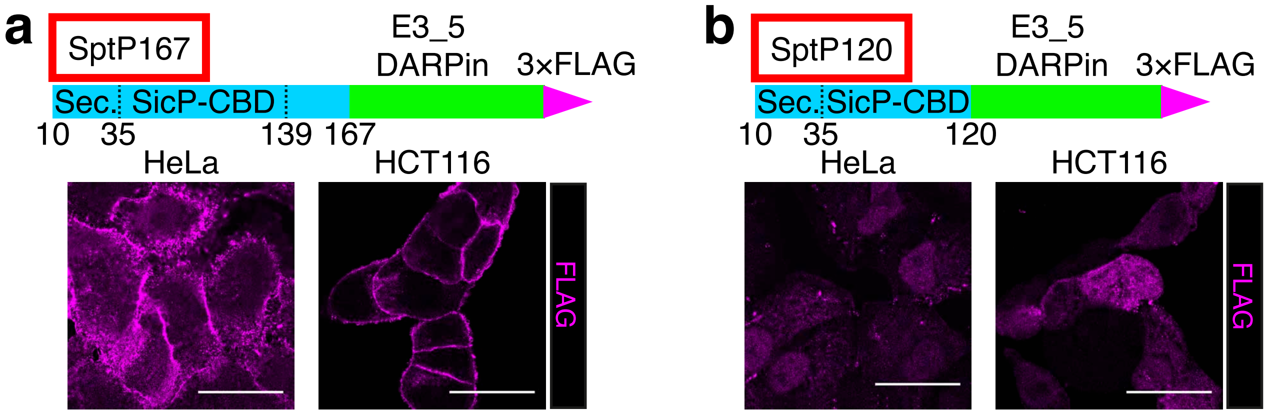

Supplementary Figure 2. SptP167-induced membrane localization of the delivered E3_5 control DARPin.
a,b, (Top) Schematic of the transferred SptP167 and SptP120 3xFLAG-tagged E3_5 Control DARPin. Secretion signal (Sec.), SicP-chaperone binding domain (SicP-CBD) and amino acid numbers that mark the beginning and end of each SptP domain are indicated. Representative anti-FLAG-immunostaining images of E3_5 Control FLAG-tagged DARPins transferred into HeLa and HCT116 cells using the SptP167 (**a**) or SptP120 (**b**) secretion tags (1 hour infection at a MOI of 100). Scale bars represent 25 μm.

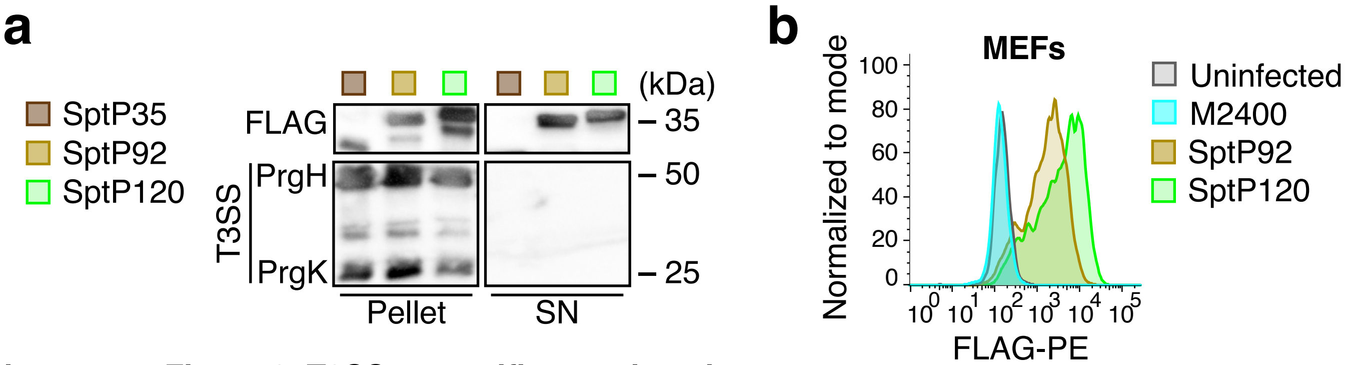

Supplementary Figure 3. T3SS-1 specific translocation.
a, Anti-FLAG tag Western blot of bacterial pellets and supernatants (SN) of arabinose induced Salmonella strain M2400[1] transformed with pCASP-HilA encoding FLAG-tagged HA4-7c12 Tandem Monobody[2] fused to SptP35 (braun), SptP92 (khaki) or SptP120 (green). Anti-needle complex (T3SS) blot serves as a control for presence of Salmonella. The expected size of the 3 different fusion proteins are about 28, 35 and 38 kDa. Note that the SptP mRNA sequence encoding the first nine amino acids is not translated because of the mRNA structure[3]. **b,** Flow cytometry analysis of FLAG-tagged HA4-7c12 Tandem Monobody fused to SptP92 (khaki) or SptP120 (green) and transferred into HeLa cells following 1 hour infection at a MOI of 100. Uninfected cells and Salmonella strain M2400 with an empty pCASP-HilA vector (M2400) serve as negative control. Experiments were repeated in two biological replicates showing comparable results.

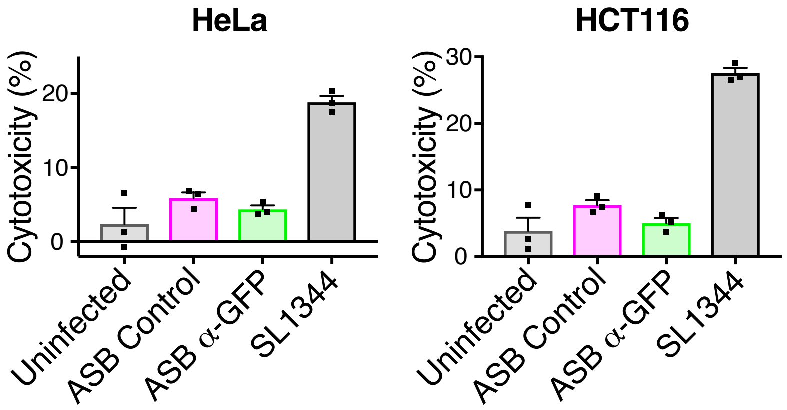

Supplementary Figure 4. Salmonella ASB2519 is an avirulent protein delivery system.
Cytotoxicity was assessed in HeLa and HCT116 cells infected for 3 hours at a MOI of 100 with Salmonella ASB2519 delivering control (ASB Control) or anti-GFP (ASB α-GFP) SptP120-DARPins. Uninfected HeLa or HCT116 cells and wild-type S. typhimurium (SL1344)[4] infection served as negative and positive controls, respectively. Cytotoxicity was determined using the Lactate dehydrogenase (LDH) release CytoTox 96 Non-Radioactive assay. Data represent the mean ± SEM of three technical replicates. Individual data points are shown.

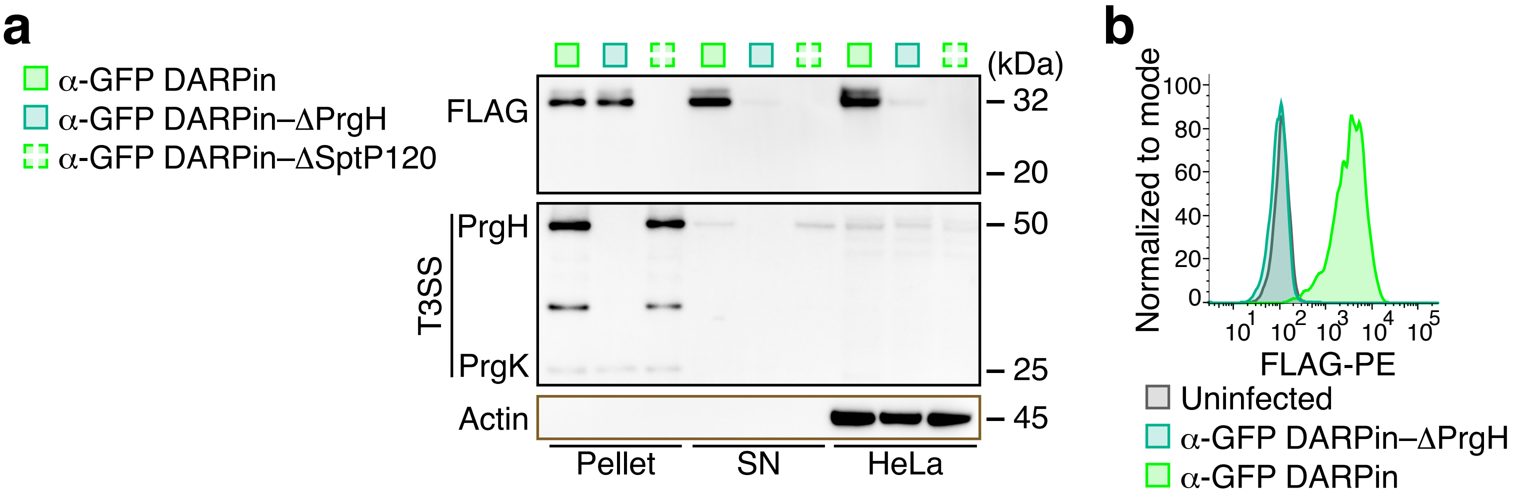

Supplementary Figure 5. T3SS-1 specific translocation.
a, Anti-FLAG tag Western blot of bacterial pellets and supernatants (SN) of arabinose induced Salmonella ASB2519 (green) and ASB2519 bearing a prgH knock-out (cyan) both transformed with pCASP-HilA encoding SptP120-α-GFP 3G124 DARPin as well as ASB2519 transformed with the same plasmid lacking SptP120 (white crossed green) resulting in impaired expression of the construct. HeLa cells infected with those Salmonella for 1 hour at a MOI of 100 were probed for transfer of protein. The anti-needle complex (T3SS) blot serves to validate the PrgH knock-out and presence of Salmonella (PrgK) while the anti-Actin blot serves as eukaryotic cells loading control. **b,** Flow cytometry analysis of FLAG-tagged SptP120-α-GFP 3G124 DARPin transferred into HeLa cells following 1 hour infection at a MOI of 100 using either ASB2519 (green) or ASB2519 mutated for PrgH (cyan). Uninfected cells serve as negative control. Experiments were repeated in two technical replicates showing comparable results.

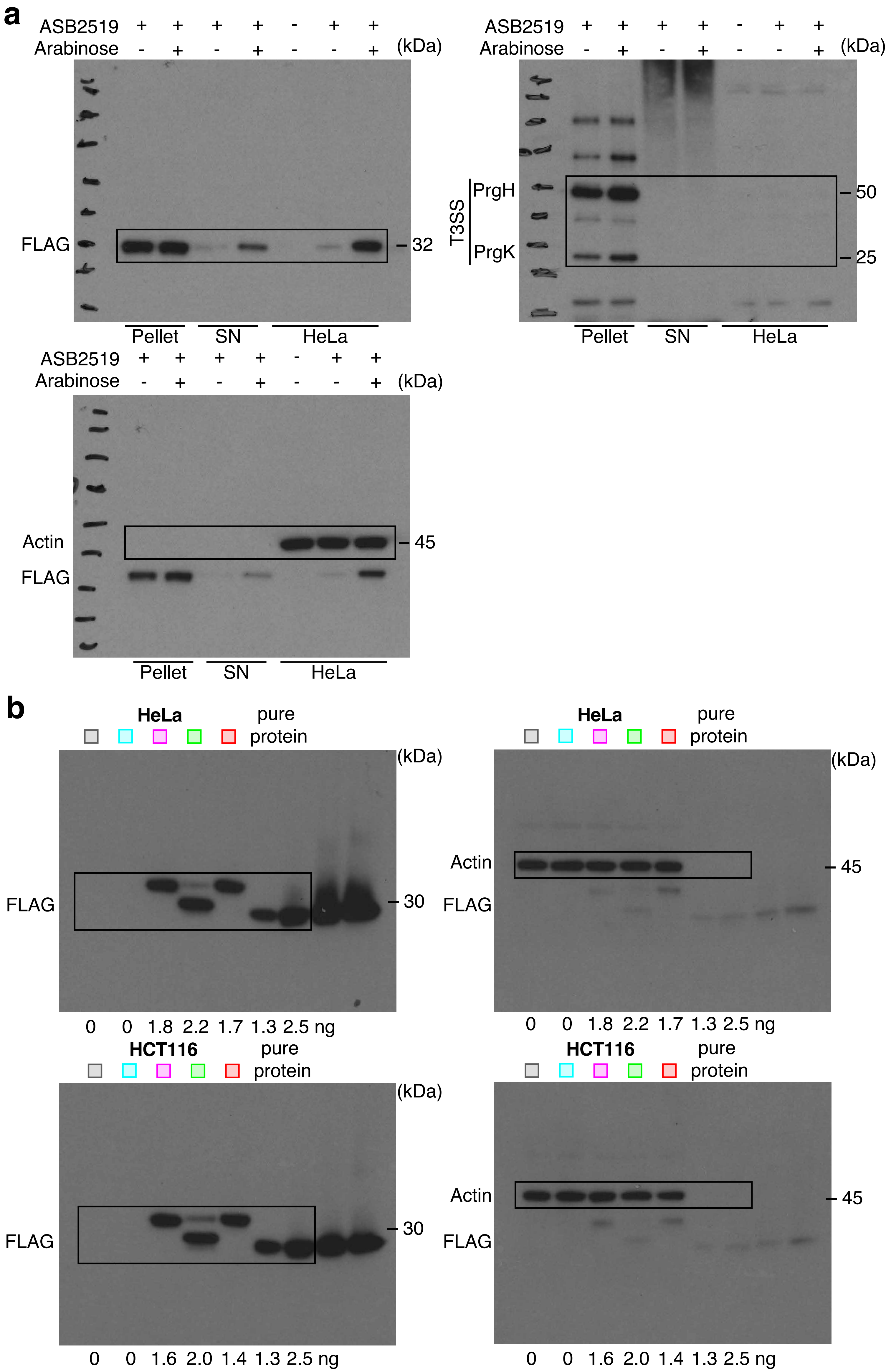

Supplementary Figure 6. Uncropped western blot images presented in main Figure 1.
a, Uncropped blots of Fig. 1b. **b,** Uncropped blots of Fig. 1f. Anti-FLAG and anti-Actin antibodies were subsequently blotted on the same membranes.

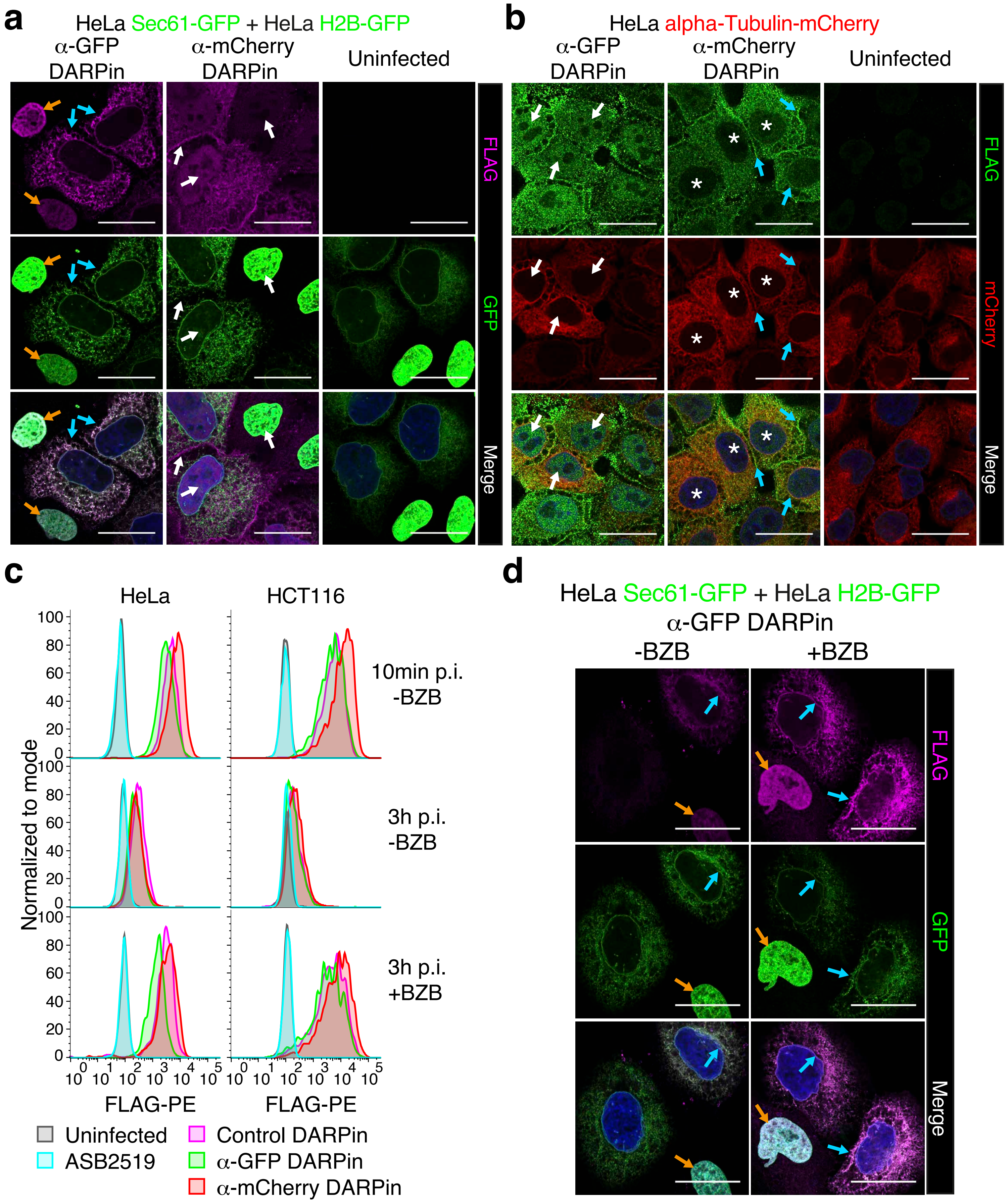

Supplementary Figure 7. Delivered SptP120-DARPin fusion proteins are functional.
a,b, Representative anti-FLAG-immunostainings of anti-GFP (α-GFP) and α-mCherry FLAG-tagged SptP120-DARPins transferred into HeLa cells expressing either Sec61-GFP (blue arrows) or H2B-GFP (orange arrows) (**a**) or alpha-Tubulin-mCherry (**b**). Note in (**b**) the absence of α-mCherry FLAG-tagged SptP120-DARPins in nuclei because they bind their respective target in the cytoplasm, indicated with asterisks. **c,** Flow cytometry analysis of the indicated FLAG-tagged SptP120-DARPins transferred into HeLa and HCT116 cells (1-hour infection, MOI of 100) and analyzed 10 minutes (upper histograms) or 3 hours post-infection (p.i.), with (lower histograms) or without (middle histograms) bortezomib (BZB, 50 nM). Uninfected cells and Salmonella ASB2519 with an empty pCASP-HilA vector (ASB2519) are shown as negative controls. **d,** Representative anti-FLAG-immunostainings of the α-GFP FLAG-tagged SptP120-DARPin transferred into HeLa cells expressing Sec61-GFP or H2B-GFP, analyzed 3 hours post-infection with or without BZB. Experiments were repeated in three biological replicates showing comparable results. Scale bars are 25 μm. Blue and orange arrows indicate colocalization in the cytoplasm and nuclei, respectively. White arrows show that the FLAG-tagged DARPins do not cross co-localize with proteins fused to domains other than their target (GFP (**a**) or mCherry (**b**)), demonstrating specificity.

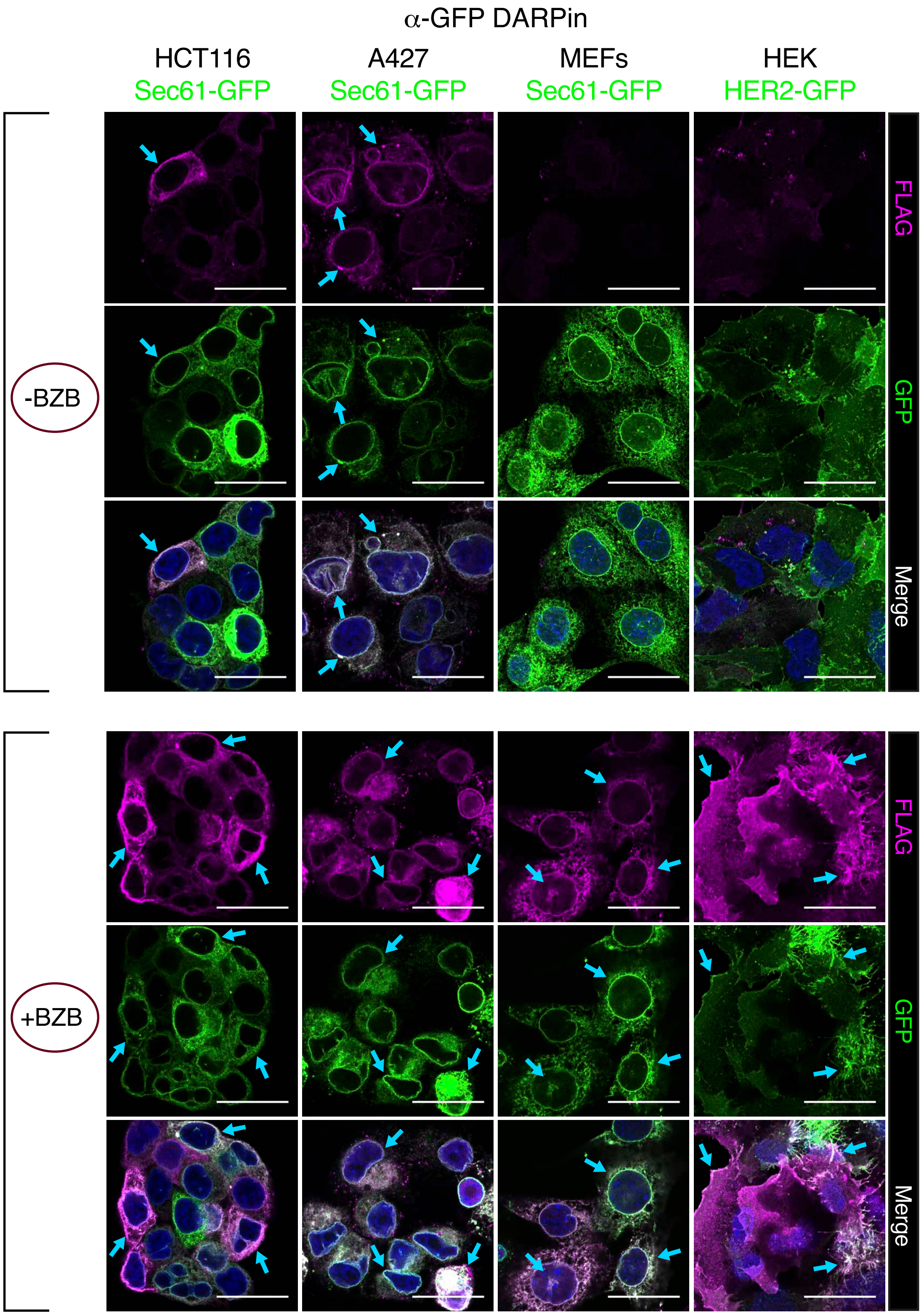

Supplementary Figure 8. Functional delivery of SptP120-DARPin fusion proteins into multiple cell types.

Representative anti-FLAG-immunostainings of the anti-GFP (α-GFP) FLAG-tagged SptP120-DARPin transferred into the indicated cells (1 hour infection at a MOI of 100) and analyzed 3 hours post-infection without (upper panels) or with (lower images) bortezomib (BZB, 50 nM) Experiments were performed in HCT116, A427 and MEF cells expressing Sec61-GFP as well as in HEK293 cells expressing HER2-GFP. Experiments were repeated in three biological replicates showing comparable results. Scale bars are 25 μm. Blue arrows indicate cytoplasmic colocalization of the α-GFP SptP120-DARPin with the GFP-tagged target proteins.

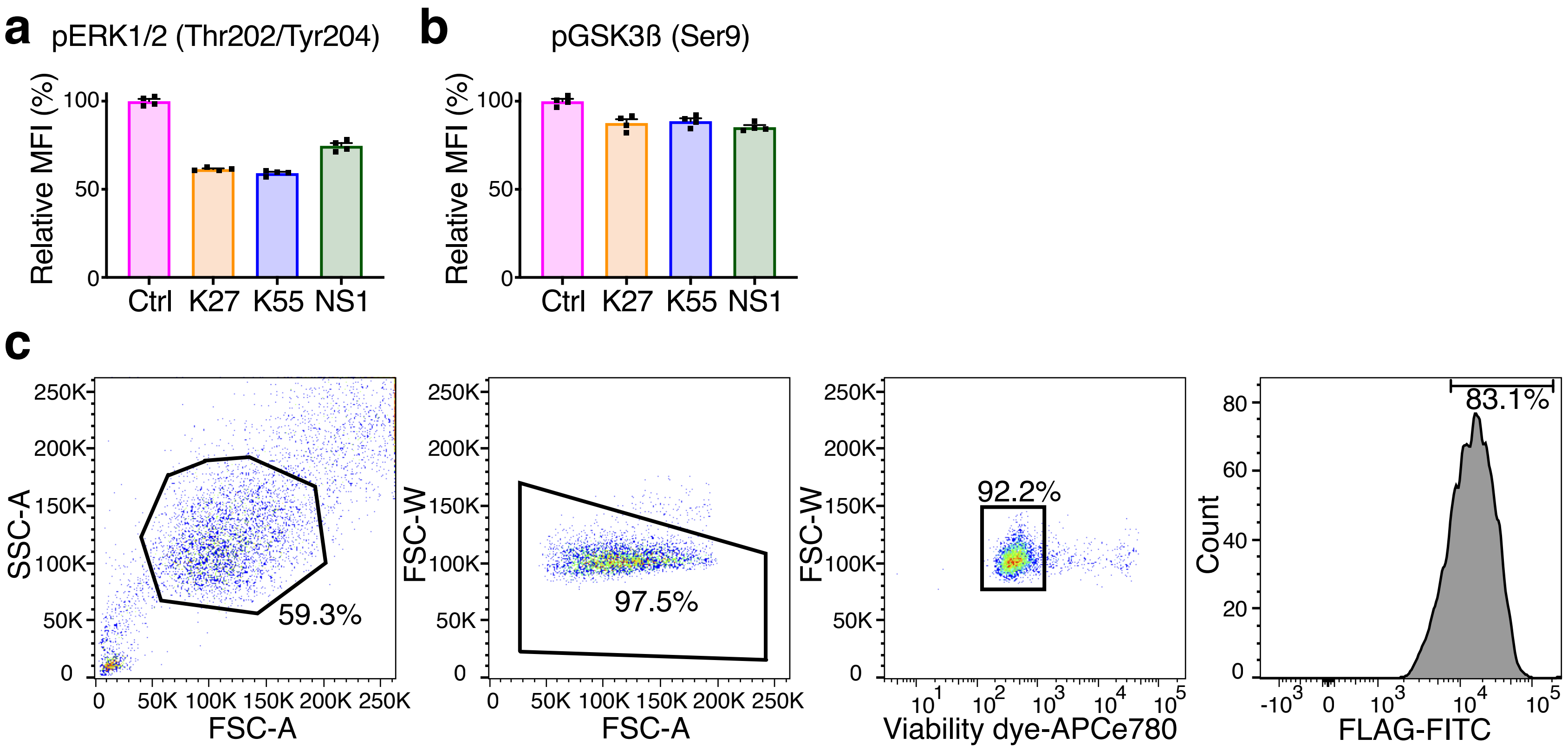

Supplementary Figure 9. Transferred SptP120-RAS inhibitors downregulate KRASG13D activation.
a, b, Flow cytometric measurements of ERK1/2 phosphorylation in HCT116 cells upon FLAG-positive delivery of the indicated SptP120-anti-RAS binders. Data were analysed 3 hours post-infection in the presence of bortezomib (BZB, 50 nM). Data are shown as relative median fluorescence intensities (MFI) ± SEM of ERK1/2 (**a**) and GSK3β (**b**) phosphorylation, compared to SptP120-control DARPin (Ctrl) treated cells. Data represent two biological replicates measured in duplicates. Individual data points are shown. **c,** Representative example of the gating strategy used for flow cytometry analysis of ERK1/2 and GSK3β phosphorylation in Fig. 4 and Supplementary Fig. 9.

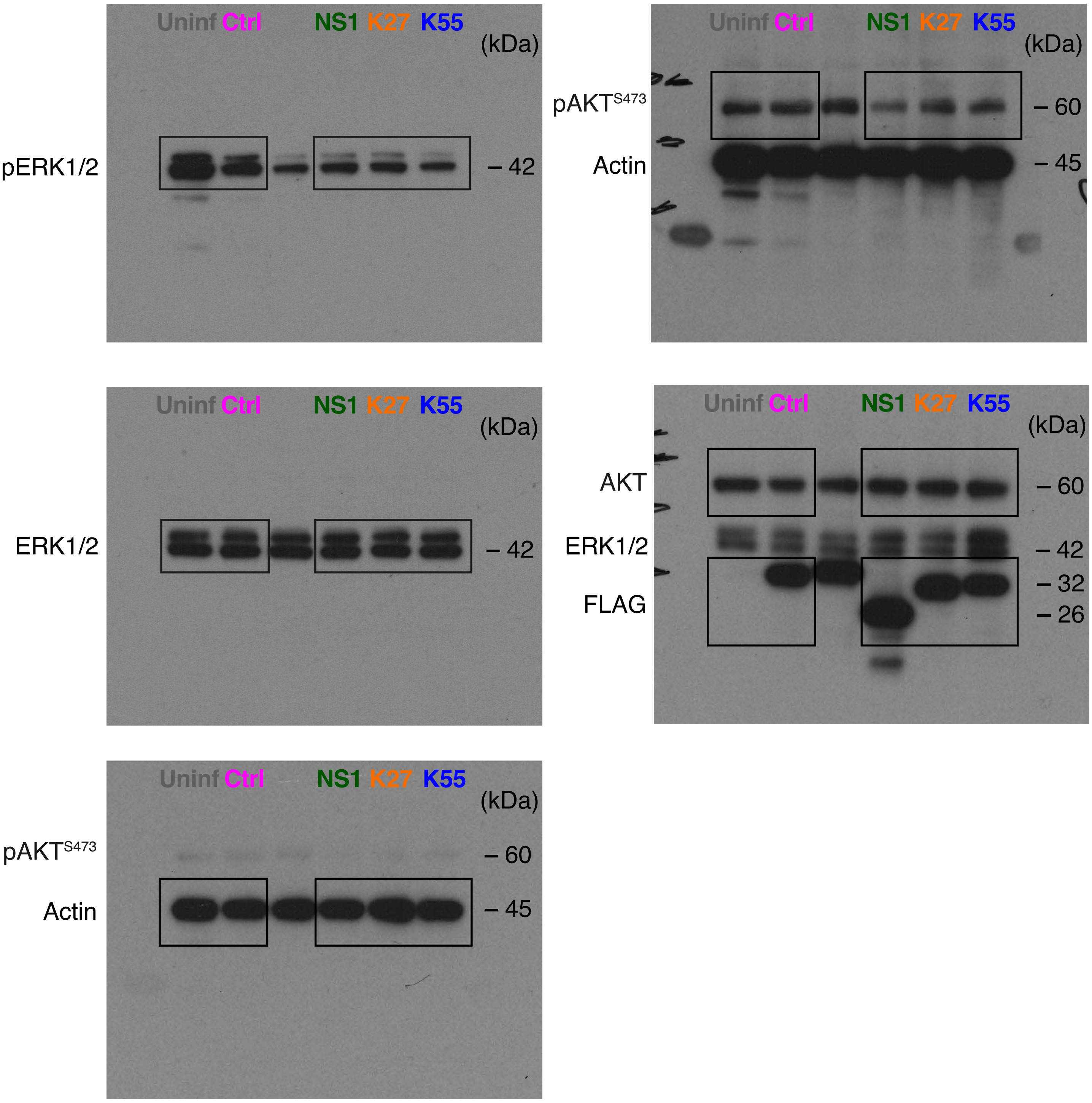

Supplementary Figure 10. Uncropped western blot images presented in main Figure 4. Uncropped blots of Fig. 4a. Anti-pERK1/2 and anti-ERK1/2 antibodies were blotted first. Due to different protein sizes, membranes were then subsequently blotted either with anti-pAKT^{S473} and anti-Actin antibodies or anti-AKT and anti-FLAG antibodies together.

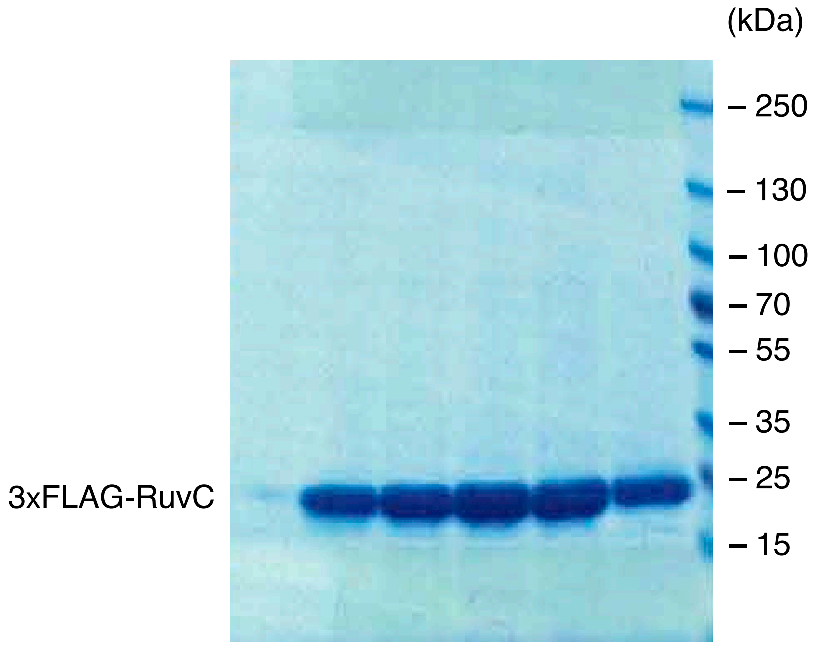

Supplementary Figure 11. Purity of 3xFLAG-RuvC protein.
3xFLAG-RuvC protein peak fractions from a Superdex 200 size exclusion column were analyzed via SDS-PAGE followed by Coomassie blue staining. Pure 3xFLAG-RuvC protein was used as quantification reference in main Fig. 1f.

Supplementary Tables

Supplementary Table 1. List of strains used in this study

Salmonella typhimurium Strain	Genotype	References
SL1344	wild-type	[4]
SB2519	ΔSipA ΔSptP ΔAvrA ΔSopE ΔSopE2 ΔSipF ΔSopB ΔSopD ΔSopD2 ΔSlrP ΔGtgE	[5]
ASB2519	ΔSipA ΔSptP ΔAvrA ΔSopE ΔSopE2 ΔSipF ΔSopB ΔSopD ΔSopD2 ΔSlrP ΔGtgE ΔSopA ΔSsaK	This study
HSB2519	ΔSipA ΔSptP ΔAvrA ΔSopE ΔSopE2 ΔSipF ΔSopB ΔSopD ΔSopD2 ΔSlrP ΔGtgE ΔSopA ΔSsaK ΔPrgH	This study
M2400	ΔSipA ΔSptP ΔSopE ΔSopE2 ΔSopB ΔSopA ΔSpvB ΔSpvC	[1]

Supplementary Table 2. Primer sequences

Primer	5' to 3' forward sequence
ACp100	GGACAAATCCGCCGCCCTAGACCGATGCATAATGTGCCTGTCA
ACp101	TCGCCGCAGCCGAACGCCCTAGACTCAGGAGAGCGTTCACCGA
ACp133	GTCGACGATTATAAAGATCATG
ACp134	ACTTTCTGCTCCAACATCGTTA
ACp145	AGTATTTTCCTTAGCAATATAA
ACp146	CCCATATTTTTCCGTTAGTGCA
ACp147	CGTGATCTGCACTGCTAAACGT
ACp248	CATGATCTTTATAATCGTCGACGGCTGCCTTTTGCAGGACTTCA
ACp255	TAAGGCGTTAAAAATCCAGACCGTTTTTCCATAATGATGTTGTAGGCTGGAGCTGCTTCGAA
ACp256	TTCCATGCGGGTTGAGGCTGGACTACGCCCAGGCCAGTGGCATATGAATATCCTCCTTAGTT
ACp258	GTATACTTTGGCCGAAGACTTCTCTTACCGAGATTATCTTTGTAGGCTGGAGCTGCTTCGAA
ACp259	CATTTTTATCTCATTAATTTTAATATTCATCGCTACCTCTCATATGAATATCCTCCTTAGTT
ACp292	ATTCCTGCAGTATGTTTTTGAG
ACp326	CGATCCAGTGTGCTGGAATTAA
ACp327	GGTGGCCTCCTGTGTTCTGGCG
ACp328	CGCCAGAACACAGGAGGCCACCATGGTGAGCAAGGGCGAGGAGG
ACp329	CAGTCATAGATCTGAGTCCGGACTTGTACAGCTCGTCCATGCCG
ACp330	GTACAAGTCCGGACTCAGATCTATGACTGAATATAAACTTGTGG
ACp331	TTAATTCCAGCACACTGGATCGTTACATAATTACACACTTTGTC
ACp367	GCGTTTGTAGATTATCAACGCCCGTATCAAATTTTGCTGATGTAGGCTGGAGCTGCTTCGAA
ACp368	CACTTTTCATTCTATTTTCATCAGGAATCCCTGTGTCCTGCATATGAATATCCTCCTTAGTT
ACp370	CTCAAAAACATACTGCAGGAATGACCTGGGCAAAAAATTGCTTG

Supplementary Table 3. List of plasmids used in this study

**Plasmid**	**Description**
pCASP-SptP167-3G124-HilA	triggers secretion of SptP167 fused to α-GFP DARPin from *Salmonella* upon induction with arabinose
pCASP-SptP120-3G124-HilA	triggers secretion of SptP120 fused to α-GFP DARPin from *Salmonella* upon induction with arabinose
pCASP-3G124-HilA	expression of α-GFP DARPin from *Salmonella* is disrupted
pCASP-SptP120-3m160-HilA	triggers secretion of SptP120 fused to α-mCherry DARPin from *Salmonella* upon induction with arabinose
pCASP-SptP120-E3_5-HilA	triggers secretion of SptP120 fused to E3_5 DARPin from *Salmonella* upon induction with arabinose
pCASP-SptP120-NS1-HilA	triggers secretion of SptP120 fused to NS1 monobody from *Salmonella* upon induction with arabinose
pCASP-SptP120-K27-HilA	triggers secretion of SptP120 fused to K27 DARPin from *Salmonella* upon induction with arabinose
pCASP-SptP120-K55-HilA	triggers secretion of SptP120 fused to K55 DARPin from *Salmonella* upon induction with arabinose
pCASP-SptP120-TD-HilA	triggers secretion of SptP120 fused to HA4-7c12 Tandem Monobody from *Salmonella* upon induction with arabinose
pCASP-SptP92-TD-HilA	triggers secretion of SptP92 fused to HA4-7c12 Tandem Monobody from *Salmonella* upon induction with arabinose
pCASP-SptP35-TD-HilA	triggers secretion of SptP35 fused to HA4-7c12 Tandem Monobody from *Salmonella* upon induction with arabinose
pCASP-HilA empty vector	triggers HilA overexpression upon induction with arabinose without any synthetic protein expressed from *Salmonella*
pCMV R8.74	lentiviral packaging plasmid – gift from Didier Trono (Addgene plasmid # 22036)
pMD2.G	vesicular stomatitis G envelope expressing plasmid – gift from Dider Trono (Addgene plasmid # 12259)
Lenti-AcGFP-Sec61-IRES-Blast	vector plasmid for integration and constitutive expression of AcGFP fused to Sec61 in mammalian cells – gift from D. Gerlich (IMBA, Vienna).
Lenti-mCherry-WT-KRAS-IRES-Blast	vector plasmid for integration and constitutive expression of mCherry fused to wild-type KRAS in mammalian cells
Lenti-mCherry-G12V-KRAS-IRES-Blast	vector plasmid for integration and constitutive expression of mCherry fused to $KRAS^{G12V}$ in mammalian cells

All plasmid maps and sequences can be found on Addgene.org

Supplementary Table 4. List of cell lines used in this study

Cell line	Description
HCT116	Human colorectal carcinoma derived cell line bearing a $KRAS^{G13D}$ activating mutation – obtained from ATCC
HCT116<br>G12V KRAS-mCherry	HCT116 cells engineered to stably express mutated G12V KRAS fused to mCherry – created in this study by lentiviral transduction
HCT116<br>Sec61-GFP	HCT116 cells engineered to stably express Sec61 fused to AcGFP – created in this study by lentiviral transduction
HeLa	HeLa Kyoto – human cervix adenocarcinoma derived cell line – gift from the lab of D. Gerlich (IMBA, Vienna)
HeLa<br>Sec61-GFP	HeLa Kyoto cells engineered to stably express Sec61 fused to AcGFP – gift from the lab of D. Gerlich (unpublished)
HeLa<br>H2B-GFP	HeLa Kyoto cells engineered to stably express H2B fused to EGFP – gift from the lab of D. Gerlich [ref. 6]
HeLa<br>alpha-Tubulin-mCherry	HeLa Kyoto cells engineered to stably express alpha-Tubulin fused to mCherry – gift from the lab of D. Gerlich [ref. 6]
HeLa<br>Sec61-GFP<br>H2B-mCherry	HeLa Kyoto cells engineered to stably express Sec61 fused to AcGFP and H2B fused to mCherry – created in this study by lentiviral transduction of HeLa Kyoto cells that also stably express H2B-mCherry. The H2B-mCherry cells were a gift from D. Gerlich
HeLa<br>WT KRAS-mCherry	HeLa Kyoto cells engineered to stably express wild-type KRAS fused to mCherry – created in this study by lentiviral transduction
A427<br>Sec61-GFP	Human lung carcinoma derived cell line engineered to stably express Sec61 fused to AcGFP – created in this study by lentiviral transduction
MEFs<br>Sec61-GFP	Immortalized mouse embryonic fibroblast (MEFs) engineered to stably express Sec61 fused to AcGFP – created in this study by lentiviral transduction of immortalized MEFs. Immortalized MEFs were a gift from the laboratory of J. Zuber (IMP, Vienna).
HEK<br>HER2-GFP	HEK293 – human embryonic kidney cells derived cell line engineered to stably express HER2 fused to GFP

Supplementary references

1. Hoffmann, C. *et al.* In macrophages, caspase-1 activation by SopE and the type III secretion system-1 of S. typhimurium can proceed in the absence of flagellin. *PLoS One* **5**, e12477 (2010).
2. Grebien, F. *et al.* Targeting the SH2-kinase interface in Bcr-Abl inhibits leukemogenesis. *Cell* **147**, 306–319 (2011).
3. Button, J. E. & Galán, J. E. Regulation of chaperone/effector complex synthesis in a bacterial type III secretion system. *Mol. Microbiol.* **81**, 1474–1483 (2011).
4. Hoiseth, S. K. & Stocker, B. A. Aromatic-dependent Salmonella typhimurium are non-virulent and effective as live vaccines. *Nature* **291**, 238–9 (1981).
5. Spano, S., Gao, X., Hannemann, S., Lara-Tejero, M. & Galan, J. E. A Bacterial Pathogen Targets a Host Rab-Family GTPase Defense Pathway with a GAP. *Cell Host Microbe* **19**, 216–226 (2016).
6. Steigemann, P. *et al.* Aurora B-Mediated Abscission Checkpoint Protects against Tetraploidization. *Cell* **136**, 473–484 (2009).
