# Supplementary material for: Salmonella-based platform for efficient delivery of functional binding proteins to the cytosol
Source: Commun Biol. 2020 Jul 3;3:342. doi: 10.1038/s42003-020-1072-4 (PMC7335062; doi:10.1038/s42003-020-1072-4)
Supplement: Supplementary file 2 — Supplementary Data 1 [file 42003_2020_1072_MOESM2_ESM.pdf]

	Cells/Single Cells/Live/FLAG+ Median (PE-A)	Relative MFI
AC20180531_pERK_HCT116_E3_5+BZB	2189	100
AC20180531_pERK_HCT116_K27+BZB	1506	68.7985381
AC20180531_pERK_HCT116_K55+BZB	1345	61.4435815
AC20180531_pERK_HCT116_NS1+BZB	1335	60.9867519
AC20180606_pERK_HCT116_E3_5+BZB	2723	100
AC20180606_pERK_HCT116_K27+BZB	2025	74.3665075
AC20180606_pERK_HCT116_K55+BZB	1642	60.3011385
AC20180606_pERK_HCT116_NS1+BZB	1731	63.5695924
AC20180608_pERK_HCT116_E3_5+BZB	2639	100
AC20180608_pERK_HCT116_K27+BZB	1815	68.7760515
AC20180608_pERK_HCT116_K55+BZB	1748	66.2372111
AC20180608_pERK_HCT116_NS1+BZB	1953	74.005305
AC20180629_pERK_HCT116_E3_5+BZB	1899	100
AC20180629_pERK_HCT116_K27+BZB	1368	72.0379147
AC20180629_pERK_HCT116_K55+BZB	1141	60.0842549
AC20180629_pERK_HCT116_NS1+BZB	1147	60.4002106
AC20180706_pERK_HCT116_E3_5+BZB	2103	100
AC20180706_pERK_HCT116_K27+BZB	1320	62.767475
AC20180706_pERK_HCT116_K55+BZB	1244	59.1535901
AC20180706_pERK_HCT116_NS1+BZB	1397	66.4289111
AC20180725_pERK_HCT116_E3_5+BZB	2359	100
AC20180725_pERK_HCT116_K27+BZB	1487	63.0351844
AC20180725_pERK_HCT116_K55+BZB	1239	52.5222552
AC20180725_pERK_HCT116_NS1+BZB	1302	55.1928783

Supplementary Data 1
