# Supplementary material for: Salmonella-based platform for efficient delivery of functional binding proteins to the cytosol
Source: Commun Biol. 2020 Jul 3;3:342. doi: 10.1038/s42003-020-1072-4 (PMC7335062; doi:10.1038/s42003-020-1072-4)
Supplement: Supplementary file 3 — Supplementary Data 2 [file 42003_2020_1072_MOESM3_ESM.pdf]

	Cells/Single Cells/Live/FLAG+ Median (Pacific Blue-A)	Relative MFI
AC20180531_pGSK3b_HCT116_E3_5+BZB	8562	100
AC20180531_pGSK3b_HCT116_K27+BZB	7333	85.64587713
AC20180531_pGSK3b_HCT116_K55+BZB	6821	79.6659659
AC20180531_pGSK3b_HCT116_NS1+BZB	6573	76.76944639
AC20180606_pGSK3b_HCT116_E3_5+BZB	11586	100
AC20180606_pGSK3b_HCT116_K27+BZB	10483	90.47988952
AC20180606_pGSK3b_HCT116_K55+BZB	9620	83.03124461
AC20180606_pGSK3b_HCT116_NS1+BZB	9054	78.14603832
AC20180608_pGSK3b_HCT116_E3_5+BZB	10019	100
AC20180608_pGSK3b_HCT116_K27+BZB	8486	84.69907176
AC20180608_pGSK3b_HCT116_K55+BZB	8448	84.31979239
AC20180608_pGSK3b_HCT116_NS1+BZB	8081	80.65675217
AC20180629_pGSK3b_HCT116_E3_5+BZB	8834	100
AC20180629_pGSK3b_HCT116_K27+BZB	7284	82.4541544
AC20180629_pGSK3b_HCT116_K55+BZB	7496	84.85397329
AC20180629_pGSK3b_HCT116_NS1+BZB	6866	77.72243604
AC20180706_pGSK3b_HCT116_E3_5+BZB	10602	100
AC20180706_pGSK3b_HCT116_K27+BZB	8448	79.68307866
AC20180706_pGSK3b_HCT116_K55+BZB	7714	72.75985663
AC20180706_pGSK3b_HCT116_NS1+BZB	7748	73.08055084
AC20180725_pGSK3b_HCT116_E3_5+BZB	10699	100
AC20180725_pGSK3b_HCT116_K27+BZB	9555	89.30741191
AC20180725_pGSK3b_HCT116_K55+BZB	8893	83.11991775
AC20180725_pGSK3b_HCT116_NS1+BZB	8486	79.31582391

Supplementary Data 2
