# Supplementary material for: Salmonella-based platform for efficient delivery of functional binding proteins to the cytosol
Source: Commun Biol. 2020 Jul 3;3:342. doi: 10.1038/s42003-020-1072-4 (PMC7335062; doi:10.1038/s42003-020-1072-4)
Supplement: Supplementary file 4 — Supplementary Data 3 [file 42003_2020_1072_MOESM4_ESM.pdf]

	Cells/Single Cells/Live/FLAG+ Median (PE-A)	Relative MFI
AC20180629_pERK_HK_E3_5+BZB_noEGF	2270	43.1805212
AC20180629_pERK_HK_E3_5+BZB_20ngEGF	5257	100
AC20180629_pERK_HK_K27+BZB_noEGF	2083	39.6233593
AC20180629_pERK_HK_K27+BZB_20ngEGF	2495	47.4605288
AC20180629_pERK_HK_K55+BZB_noEGF	1558	29.6366749
AC20180629_pERK_HK_K55+BZB_20ngEGF	2849	54.1944075
AC20180629_pERK_HK_NS1+BZB_noEGF	2270	43.1805212
AC20180629_pERK_HK_NS1+BZB_20ngEGF	4343	82.613658
AC20180706_pERK_HK_E3_5+BZB_noEGF	2828	47.5613858
AC20180706_pERK_HK_E3_5+BZB_20ngEGF	5946	100
AC20180706_pERK_HK_K27+BZB_noEGF	2660	44.7359569
AC20180706_pERK_HK_K27+BZB_20ngEGF	2759	46.4009418
AC20180706_pERK_HK_K55+BZB_noEGF	2203	37.0501177
AC20180706_pERK_HK_K55+BZB_20ngEGF	2853	47.9818365
AC20180706_pERK_HK_NS1+BZB_noEGF	2734	45.9804911
AC20180706_pERK_HK_NS1+BZB_20ngEGF	4026	67.7093845
AC20180725_pERK_HK_E3_5+BZB_noEGF	1642	39.3199234
AC20180725_pERK_HK_E3_5+BZB_20ngEGF	4176	100
AC20180725_pERK_HK_K27+BZB_noEGF	1646	39.4157088
AC20180725_pERK_HK_K27+BZB_20ngEGF	1603	38.3860153
AC20180725_pERK_HK_K55+BZB_noEGF	1532	36.6858238
AC20180725_pERK_HK_K55+BZB_20ngEGF	2029	48.5871648
AC20180725_pERK_HK_NS1+BZB_noEGF	1549	37.0929119
AC20180725_pERK_HK_NS1+BZB_20ngEGF	2917	69.8515326

Supplementary Data 3
