# Supplementary material for: Salmonella-based platform for efficient delivery of functional binding proteins to the cytosol
Source: Commun Biol. 2020 Jul 3;3:342. doi: 10.1038/s42003-020-1072-4 (PMC7335062; doi:10.1038/s42003-020-1072-4)
Supplement: Supplementary file 5 — Supplementary Data 4 [file 42003_2020_1072_MOESM5_ESM.pdf]

| | Cells/Single Cells/Live/FLAG+ Median (Pacific Blue-A) | Relative MFI |
|---|---|---|
| AC20180629_pGSK3b_HK_E3_5+BZB_noEGF | 8697 | 77.3204125 |
| AC20180629_pGSK3b_HK_E3_5+BZB_20ngEGF | 11248 | 100 |
| AC20180629_pGSK3b_HK_K27+BZB_noEGF | 8893 | 79.0629445 |
| AC20180629_pGSK3b_HK_K27+BZB_20ngEGF | 9364 | 83.2503556 |
| AC20180629_pGSK3b_HK_K55+BZB_noEGF | 8581 | 76.2891181 |
| AC20180629_pGSK3b_HK_K55+BZB_20ngEGF | 8814 | 78.3605974 |
| AC20180629_pGSK3b_HK_NS1+BZB_noEGF | 8736 | 77.6671408 |
| AC20180629_pGSK3b_HK_NS1+BZB_20ngEGF | 10675 | 94.905761 |
| | | |
| AC20180706_pGSK3b_HK_E3_5+BZB_noEGF | 10179 | 81.2759502 |
| AC20180706_pGSK3b_HK_E3_5+BZB_20ngEGF | 12524 | 100 |
| AC20180706_pGSK3b_HK_K27+BZB_noEGF | 9599 | 76.6448419 |
| AC20180706_pGSK3b_HK_K27+BZB_20ngEGF | 10156 | 81.0923028 |
| AC20180706_pGSK3b_HK_K55+BZB_noEGF | 9054 | 72.2931971 |
| AC20180706_pGSK3b_HK_K55+BZB_20ngEGF | 10202 | 81.4595976 |
| AC20180706_pGSK3b_HK_NS1+BZB_noEGF | 10699 | 85.4279783 |
| AC20180706_pGSK3b_HK_NS1+BZB_20ngEGF | 12553 | 100.231555 |
| | | |
| AC20180725_pGSK3b_HK_E3_5+BZB_noEGF | 8336 | 83.0196196 |
| AC20180725_pGSK3b_HK_E3_5+BZB_20ngEGF | 10041 | 100 |
| AC20180725_pGSK3b_HK_K27+BZB_noEGF | 7974 | 79.414401 |
| AC20180725_pGSK3b_HK_K27+BZB_20ngEGF | 8373 | 83.3881088 |
| AC20180725_pGSK3b_HK_K55+BZB_noEGF | 7992 | 79.593666 |
| AC20180725_pGSK3b_HK_K55+BZB_20ngEGF | 8562 | 85.2703914 |
| AC20180725_pGSK3b_HK_NS1+BZB_noEGF | 8373 | 83.3881088 |
| AC20180725_pGSK3b_HK_NS1+BZB_20ngEGF | 9427 | 93.8850712 |

**Supplementary Data 4**
