# Supplementary material for: Salmonella-based platform for efficient delivery of functional binding proteins to the cytosol
Source: Commun Biol. 2020 Jul 3;3:342. doi: 10.1038/s42003-020-1072-4 (PMC7335062; doi:10.1038/s42003-020-1072-4)
Supplement: Supplementary file 6 — Description of Additional Supplementary Files [file 42003_2020_1072_MOESM6_ESM.pdf]

Descriptions of Additional Supplementary Files

Supplementary Data 1
Source data underlying Fig. 4c.
Flow cytometric median fluorescence intensities (MFI) of ERK1/2 phosphorylation in HCT116 cells upon FLAG-positive delivery of the indicated SptP120 fused binders (E3_5, K27, K55 and NS1). Data were analysed 10 minutes post-infection in the presence of bortezomib (BZB; 50 nM). Last column shows relative MFI of ERK1/2 phosphorylation calculated compared to the SptP120-E3_5 control DARPin treated cells.

Supplementary Data 2
Source data underlying Fig. 4d.
Flow cytometric median fluorescence intensities (MFI) of GSK3β phosphorylation in HCT116 cells upon FLAG-positive delivery of the indicated SptP120 fused binders (E3_5, K27, K55 and NS1). Data were analysed 10 minutes post-infection in the presence of bortezomib (BZB; 50 nM). Last column shows relative MFI of GSK3β phosphorylation calculated compared to the SptP120-E3_5 control DARPin treated cells.

Supplementary Data 3
Source data underlying Fig. 4f.
Flow cytometric median fluorescence intensities (MFI) of ERK1/2 phosphorylation in HeLa Kyoto cells upon FLAG-positive delivery of the indicated SptP120 fused binders (E3_5, K27, K55 and NS1) in the presence of bortezomib (BZB; 50 nM). Data were analysed after 10 minutes incubation in fresh medium with or without EGF induction (20ng) post-infection. Last column shows relative MFI of ERK1/2 phosphorylation calculated compared to the SptP120-E3_5 control DARPin treated cells with EGF induction.

Supplementary Data 4
Source data underlying Fig. 4g.
Flow cytometric median fluorescence intensities (MFI) of GSK3β phosphorylation in HeLa Kyoto cells upon FLAG-positive delivery of the indicated SptP120 fused binders (E3_5, K27, K55 and NS1) in the presence of bortezomib (BZB; 50 nM). Data were analysed after 10 minutes incubation in fresh medium with or without EGF (20ng) post-infection. Last column shows relative MFI of GSK3β phosphorylation calculated compared to the SptP120-E3_5 control DARPin treated cells with EGF induction.
