# Supplementary material for: Salmonella-based platform for efficient delivery of functional binding proteins to the cytosol
Source: Commun Biol. 2020 Jul 3;3:342. doi: 10.1038/s42003-020-1072-4 (PMC7335062; doi:10.1038/s42003-020-1072-4)
Supplement: Supplementary file 7 — Reporting Summary [file 42003_2020_1072_MOESM7_ESM.pdf]

nature research

Corresponding author(s): Thomas Marlovits, Josef Penninger

Last updated by author(s): Jun 2, 2020

Reporting Summary

Nature Research wishes to improve the reproducibility of the work that we publish. This form provides structure for consistency and transparency in reporting. For further information on Nature Research policies, see our Editorial Policies and the Editorial Policy Checklist.

Statistics

For all statistical analyses, confirm that the following items are present in the figure legend, table legend, main text, or Methods section.

n/a	Confirmed	
☐	☒	The exact sample size (*n*) for each experimental group/condition, given as a discrete number and unit of measurement
☐	☒	A statement on whether measurements were taken from distinct samples or whether the same sample was measured repeatedly
☐	☒	The statistical test(s) used AND whether they are one- or two-sided<br>*Only common tests should be described solely by name; describe more complex techniques in the Methods section.*
☒	☐	A description of all covariates tested
☐	☒	A description of any assumptions or corrections, such as tests of normality and adjustment for multiple comparisons
☐	☒	A full description of the statistical parameters including central tendency (e.g. means) or other basic estimates (e.g. regression coefficient) AND variation (e.g. standard deviation) or associated estimates of uncertainty (e.g. confidence intervals)
☐	☒	For null hypothesis testing, the test statistic (e.g. *F*, *t*, *r*) with confidence intervals, effect sizes, degrees of freedom and *P* value noted<br>*Give P values as exact values whenever suitable.*
☒	☐	For Bayesian analysis, information on the choice of priors and Markov chain Monte Carlo settings
☒	☐	For hierarchical and complex designs, identification of the appropriate level for tests and full reporting of outcomes
☒	☐	Estimates of effect sizes (e.g. Cohen's *d*, Pearson's *r*), indicating how they were calculated

Our web collection on statistics for biologists contains articles on many of the points above.

Software and code

Policy information about availability of computer code

Data collection	ZEN Blue Edition software V2.0 (Zeiss) was used to collect fluorescent images.<br>FACSDIVA software V8.01 (BD) was used to collect FACS data.
Data analysis	ImageJ software V1.51 was used for quantification of Western blot bands.<br>Statistical analysis was performed using GraphPad Prism version 7.00.<br>FACS data was analysed using FlowJo software V10.3.

For manuscripts utilizing custom algorithms or software that are central to the research but not yet described in published literature, software must be made available to editors and reviewers. We strongly encourage code deposition in a community repository (e.g. GitHub). See the Nature Research guidelines for submitting code & software for further information.

Data

Policy information about availability of data

All manuscripts must include a data availability statement. This statement should provide the following information, where applicable:

- Accession codes, unique identifiers, or web links for publicly available datasets
- A list of figures that have associated raw data
- A description of any restrictions on data availability

The datasets generated and analyzed during the current study are available from the corresponding authors upon reasonable request. All plasmids have been deposited to addgene with unique identifiers (ID: 153322-153333, 153335, 153336 and 154009). Associated raw data for Fig. 4c,d,f, and g can be found as supplementary data.

Field-specific reporting

Please select the one below that is the best fit for your research. If you are not sure, read the appropriate sections before making your selection.

☒ Life sciences ☐ Behavioural & social sciences ☐ Ecological, evolutionary & environmental sciences

For a reference copy of the document with all sections, see nature.com/documents/nr-reporting-summary-flat.pdf

Life sciences study design

All studies must disclose on these points even when the disclosure is negative.

Sample size	No statistical methods were used to predetermine sample size.<br>All sample sizes are indicated in the figures and/or figure legends.<br>Experiments were repeated at least 2 independent times to confirm the findings.
Data exclusions	no data were excluded
Replication	All experiments in this study were independently replicated, with biological and technical replicates listed in the legends of the corresponding figures. On the graphs individual dots represent individual samples. For each experiment, all attempts at replication were successful and our findings showed comparable results.
Randomization	does not apply
Blinding	Investigators were not blinded to allocation during experiments and outcome assessment. Blinding was not possible as the same investigator performed the experiments and analyzed the data.

Reporting for specific materials, systems and methods

We require information from authors about some types of materials, experimental systems and methods used in many studies. Here, indicate whether each material, system or method listed is relevant to your study. If you are not sure if a list item applies to your research, read the appropriate section before selecting a response.

Materials & experimental systems

n/a	Involved in the study	
☐	☒	Antibodies
☐	☒	Eukaryotic cell lines
☒	☐	Palaeontology and archaeology
☒	☐	Animals and other organisms
☒	☐	Human research participants
☒	☐	Clinical data
☒	☐	Dual use research of concern

Methods

n/a	Involved in the study	
☒	☐	ChIP-seq
☐	☒	Flow cytometry
☒	☐	MRI-based neuroimaging

Antibodies

Antibodies used	antibody, company, catalog number, dilution (method)<br>anti-FLAG clone M2, Sigma, F1804, 1:3000 (Western), 1:1000 (Immunofluorescence), 1:1000 (Flow)<br>anti-Actin clone AC-74, Sigma, A5316, 1:1000 (Western)<br>anti-Phospho-p44/42 MAPK (Erk1/2) (Thr202/Tyr204), Cell Signaling, 9101, 1:1000 (Western)<br>anti-p44/42 MAPK (Erk1/2), Cell Signaling, 9102, 1:1000 (Western)<br>anti-Phospho-Akt (Ser473), Cell Signaling, 9271, 1:1000 (Western)<br>anti-Akt (pan) clone 11E7, Cell Signaling, 4685, 1:1000 (Western)<br>anti-T3SS, in house generated Rabbit polyclonal, 1:3000 (Western)<br>anti-Mouse IgG H+L HRP Conjugate, Promega, W4021, 1:4000 (Western)<br>anti-Rabbit IgG HRP Linked F(ab')2, Sigma, NA9340, 1:4000 (Western)<br>anti-Mouse IgG H&L Alexa Fluor® 647, Abcam, ab150115, 1:500 (Immunofluorescence)<br>DAPI, ThermoFisher, D3571, 1:2000 (Immunofluorescence)<br>Fixable viability dye eFluor780, ThermoFisher, 65-0865, 1:1000 (Flow)<br>anti-Mouse IgG H+L F(ab')2 labelled with PE, ThermoFisher, 12-4010-87, 1:1000 (Flow)<br>anti-FLAG clone M2 labelled with FITC, Sigma, F4049, 1:50 (Flow)<br>anti-Phospho-ERK1/2 (Thr202/Tyr204) labelled with PE, Biolegend, 369506, 1:100 (Flow)<br>anti-Phospho-GSK-3β (Ser9) clone D85E12 labelled with Pacific Blue, Cell Signaling, 14310, 1:100 (Flow)
Validation	The antibodies used are commercially available and validated on their websites and used throughout research. The T3SS antibody was confirmed using specific KO Salmonella strains for specificity.

Eukaryotic cell lines

Policy information about cell lines

Cell line source(s)	The cancer cell lines HCT116 and A427 were purchased from ATCC.<br>Untransformed Hela Kyoto cells and HeLa Kyoto cells expressing either Sec61-GFP, H2B-GFP, alpha-Tubulin-mCherry or H2B-mCherry were provided by Daniel Gerlich, IMBA, Vienna.<br>Immortalized MEFs cell line was provided by Johannes Zuber, IMP, Vienna.<br>HEK293 cells expressing HER2-GFP were provided by the Plueckthun lab, University of Zurich.<br>HCT116 cells expressing Sec61-GFP or G12V KRAS-mCherry, HeLa Kyoto cells expressing Sec61-GFP and H2B-mCherry or wild-type KRAS-mCherry and A427 cells and immortalized MEFs expressing Sec61-GFP were generated in this study by lentiviral transduction of different vectors. Please refer to Supplementary Tables 3 and 4.
Authentication	Expression of all GFP and mCherry fusion proteins within cell lines was checked by FACS and correct localization was confirmed by microscopy.
Mycoplasma contamination	The cell lines used in this study were tested negative for mycoplasm.
Commonly misidentified lines<br>(See ICLAC register)	No commonly misidentified lines listed in the ICLAC database were used in this study.

Flow Cytometry

Plots

Confirm that:

☒ The axis labels state the marker and fluorochrome used (e.g. CD4-FITC).

☒ The axis scales are clearly visible. Include numbers along axes only for bottom left plot of group (a 'group' is an analysis of identical markers).

☒ All plots are contour plots with outliers or pseudocolor plots.

☒ A numerical value for number of cells or percentage (with statistics) is provided.

Methodology

Sample preparation	Please refer to Materials and Methods
Instrument	Cells were recorded on an LSR II Flow Cytometer (BD Biosciences)
Software	Data were analyzed using FlowJo v10.0.6 software (Tree Star)
Cell population abundance	N/A
Gating strategy	To gate samples for FACS analysis, cell were initially gated by FSC-A vs SSC-A for the exclusion of debris and identification of relevant population (lymphocytes) by size and granularity. For single cells, samples were further gated by FSC-A vs FSC-W. Live cells were finally gated and identified by using fixable Viability dye, eFluor780.

☒ Tick this box to confirm that a figure exemplifying the gating strategy is provided in the Supplementary Information.
